# Measures of Human Mobility Using Mobile Phone Records Enhanced with GIS Data

**DOI:** 10.1371/journal.pone.0133630

**Published:** 2015-07-20

**Authors:** Nathalie E. Williams, Timothy A. Thomas, Matthew Dunbar, Nathan Eagle, Adrian Dobra

**Affiliations:** 1 Jackson School of International Studies and Department of Sociology, University of Washington, Seattle, Washington, United States of America; 2 Department of Sociology, University of Washington, Seattle, Washington, United States of America; 3 Center for Studies in Demography and Ecology, University of Washington, Seattle, Washington, United States of America; 4 Department of Epidemiology, Harvard University, Boston, Massachusetts, United States of America; 5 Department of Statistics, Department of Biobehavioral Nursing and Health Systems, Center for Statistics and the Social Sciences, and Center for Studies in Demography and Ecology, University of Washington, Seattle, Washington, United States of America; Universitat Rovira i Virgili, SPAIN

## Abstract

In the past decade, large scale mobile phone data have become available for the study of human movement patterns. These data hold an immense promise for understanding human behavior on a vast scale, and with a precision and accuracy never before possible with censuses, surveys or other existing data collection techniques. There is already a significant body of literature that has made key inroads into understanding human mobility using this exciting new data source, and there have been several different measures of mobility used. However, existing mobile phone based mobility measures are inconsistent, inaccurate, and confounded with social characteristics of local context. New measures would best be developed immediately as they will influence future studies of mobility using mobile phone data. In this article, we do exactly this. We discuss problems with existing mobile phone based measures of mobility and describe new methods for measuring mobility that address these concerns. Our measures of mobility, which incorporate both mobile phone records and detailed GIS data, are designed to address the spatial nature of human mobility, to remain independent of social characteristics of context, and to be comparable across geographic regions and time. We also contribute a discussion of the variety of uses for these new measures in developing a better understanding of how human mobility influences micro-level human behaviors and well-being, and macro-level social organization and change.

## Introduction

Human mobility, or movement over short or long spaces for short or long periods of time, is an important yet under-studied phenomenon in the social and demographic sciences. While there have been consistent advances in understanding migration (more permanent movement patterns) and its impact on human well-being, macro-social, political, and economic organization [1–14], advances in studies of mobility have been stymied by difficulty in recording and measuring how humans move on a minute and detailed scale. There exists a reasonably large literature on mobility in the areas of urban and transportation studies, but much of it uses detailed travel surveys which are expensive to collect, have small sample sizes and limited spatial and temporal scales, are updated infrequently, and suffer from recall bias [15–17]. Consequently, studies of mobility have not yet been able to collect large scale data or widely address how differentials in mobility influence other outcomes. This gap is particularly glaring given that mobility is likely a fundamental factor in behavior and macro-level social change, with likely associations with key issues that face human societies today, including spread of infectious diseases, responses to armed conflict and natural disasters, health behaviors and outcomes, economic, social, and political well-being, and migration. In this context, new methods for measuring human mobility could lead to significant advances in the policy relevant social and demographic sciences.

Mobile phone data have recently become available for the study of human mobility. Such data are continuously collected by wireless-service providers for billing purposes and to improve the operation of their cellular networks [18]. Every time a person makes a voice call, sends a text message or goes online from their mobile phone, a call detail record (CDR) is generated which records time and day, duration and type of communication, and an identifier of the cellular tower that handled the request. The approximate spatiotemporal trajectory of a mobile phone and its user can be reconstructed by linking the CDRs associated with that phone with the locations (latitude and longitude) of the cellular towers that handled the calls. This exciting new type of data holds immense promise for studying human behavior with precision and accuracy on a vast scale never before possible with surveys or other data collection techniques [19]. As mobile phone penetration increases dramatically worldwide to an estimated 120.8 (90.2) mobile-cellular subscriptions per 100 inhabitants in developed (developing) countries by the end of 2014 [20], selection in who uses mobile phones is decreasing, thereby reducing biases related to phone ownership [21] and making CDRs ever more appropriate for studying human mobility of whole populations.

There is a significant body of literature that has already made key inroads into understanding mobility using this exciting new data source, and there have been several different measures of mobility used [21–32]. However, there has been little discussion and assessment of these measures. Consequently, we understand little about what they actually measure and how they perform. Indeed, we argue that existing measures of mobility from CDRs do not measure mobility accurately or consistently, are confounded with other contextual characteristics, and are therefore not suitable to advance mobility studies. We further argue that the need for improved measures of mobility would be best addressed immediately as this will influence the conclusions of future studies of mobility using mobile phone data.

Towards developing accurate and meaningful measures of mobility with CDRs, and advancing this promising area of social science, in this article we propose six novel measures of mobility derived from CDRs. We define key dimensions of mobility and describe existing measures of mobility and the problems they entail. Using this background, we then propose and analyze six new measures that directly address each dimension of mobility and overcome the inherent problems with existing measures by combining CDRs with detailed GIS data on road networks. We designed our measures through combining existing, tested, and effective methods from geography and mobility studies in the transportation and urban studies literature. We carefully assess our measures using CDR and GIS data from Rwanda. An important difference in our proposed measures from those used previously is that they are fundamentally based on existing spatial analytical methods, reflecting the spatial nature of mobility. A second key difference is that they account for how humans actually move, which is most often via road networks and through many places, instead of by apparition or “as the crow flies” from one place to another. A consequence of our spatial and movement perspectives is that they produce standardized measures that address only movement of humans and are not affected by other characteristics of social context besides the roads upon which people move. Another consequence is that they are designed to be broadly applicable to different geographic settings regardless of human behavioral patterns or variation in context. In many ways, our approach is simplistic in how it utilizes data and characterizes human mobility behavior. Indeed, the power of our approach rests in this simplicity, which is precisely what makes it useful in and comparable across different settings. This article ends with a discussion of the new ways in which these measures can be used to advance the scientific study of human mobility.

For illustration we analyze anonymized CDRs provided by a major cellular phone service provider in Rwanda. These data comprise all mobile phone activity in the provider’s network between June 1, 2005 and January 31, 2009 [22, 23]. To evaluate existing and new measures of mobility, we define spatiotemporal trajectories of each caller in the provider’s network from the CDRs they generate in every given month. This yields 20,139,971 person months of spatiotemporal trajectories—for additional details, see Section SI2 in S1 Supporting Information. The calculation of our mobility measures from the spatiotemporal trajectories is detailed in S1 Supporting Information, Section SI3.

## Dimensions of Mobility

In order to better define the problems with existing measures of mobility, to design new measures, and to assess measures, we delineate two key dimensions of mobility. The first key dimension is the frequency of movement, and represents the number of times a person goes anywhere. The higher the frequency, or more times a person moves, the higher should be the value of their mobility measure. What constitutes going somewhere and what designates separate trips depends on definition and these definitions vary by study and context [15, 33]. One of our primary motivations is to create coherent measures of mobility, including frequency of movement, that are meaningful in each specific context but comparable across contexts. The second key dimension of mobility is spatial range, or how far a person moves. The further a person moves, the higher should be the value of their mobility measure.

## Existing CDR-Based Measures of Mobility

Existing measures of mobility derived from CDRs include number of towers used (NTU), distance traveled-straight line (DT-SL), maximum distance traveled (MDT), and the most commonly used measure, radius of gyration (RoG)—see, among others [21–27, 29, 30]. Measures of mobility are defined with respect to a fixed period of time, e.g. hours, days, weeks, months or years. Here we chose months as the reference time period, but our methodological developments and conclusions are relevant for shorter or longer reference time periods. The NTU measure counts the number of cellular towers from which a person called in the requisite period of time. The DT-SL measure, also called average travel distance [25], is the sum of straight line or “as the crow flies” distances between towers from which consecutive calls or texts were made. The MDT measure calculates the maximum straight line distance between two towers that a person used. The RoG is determined by first finding the center of mass of all cellular towers that a person used. The straight line distances from the center of mass to each used tower are calculated, and the value of RoG is the square root of the mean of the squares of these distances. Section SI3.1 in S1 Supporting Information gives formulas and related details.

**Fig 1:**
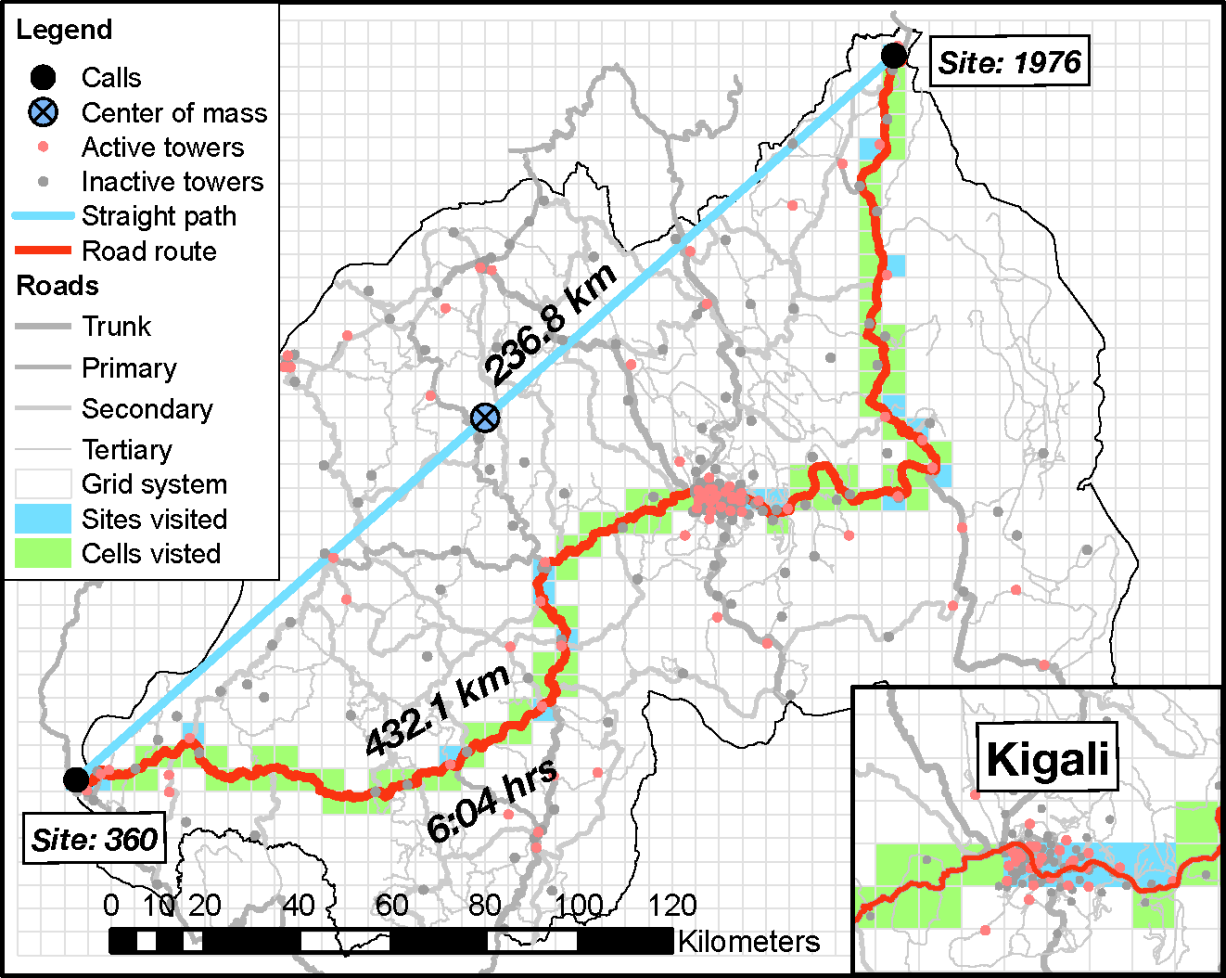
Map of the monthly spatiotemporal trajectory of the caller with the largest monthly RoG. This caller which we refer to as 𝓟 made two calls in October 2005: the first one from a cellular tower located in the grid cell labeled “Site 1976” and the second one from a cellular tower located in the grid cell labeled “Site 360.” There are 2040 5km x 5km grid cells indexed from 1 (the cell in the lower left corner) to 2040 (the cell in the upper right corner). A site is a grid cell that contains at least one cellular tower. The map shows the location of the straight path between sites 1976 and 360, and also the location of the quickest road route—the road route with the smallest estimated travel time—between the two sites. The straight path between the two towers used by 𝓟 is 236.8 km long, while the straight line path between the centroids of sites 1976 and 360 is 237.2 km long. The quickest road route between the centroids of sites 1976 and 360 is 432.1 km long. The estimated travel time along this route is 6 hours and 4 minutes. The map also shows the center of mass required for the calculation of RoG which is located in the middle of the straight path, as well as Rwanda’s borders, Rwanda’s road network structure with trunk, primary, secondary and tertiary roads, and the locations of the all the 239 cellular towers references in the Rwandan CDRs. We note that only 78 of these towers were active (i.e., handled at least one communication) in October 2005. The grid cells that contain at least one active tower in October 2005 are referred to as sites for that month. The visited sites associated with the spatiotemporal trajectory of 𝓟 are the sites which are intersected by the quickest road route between sites 1976 and 360. There are 19 visited sites which are shown in blue. All the grid cells intersected by this route are called visited cells. The visited cells that are not visited sites are shown in green. The inset shows the capital Kigali and its surrounding area. This is the region with the highest cellular tower density in Rwanda. The Rwandan road network is publicly available data under the Open Database License, and comes from OpenStreetMap (openstreetmap.org), a global open-source mapping project.

We exemplify the evaluation of these four measures with the spatiotemporal trajectory of the caller, 𝓟, who had the largest RoG from all 20 million trajectories in the Rwandan data—see Fig 1. During October 2005, 𝓟 made only two calls in this provider’s network: the first call from a location near the northern border with Uganda and the second call from a location near the western border with Democratic Republic of Congo. The NTU measure for this person is equal to 2 (10th percentile). The DT-SL and the MDT measures are both 236.8 km (78th and 100th percentile, respectively). The RoG of 𝓟 is 118.6 km.

These four measures of mobility have several critical shortcomings. Two of the primary problems are caused by their direct definition with respect to the location of the cellular towers and the fact that tower placement is not random or evenly spaced [18]. Figs 1 and A in S1 Supporting Information show the uneven variation in tower placement in Rwanda: the capital Kigali has a high tower density with respect to the rest of the country which comprises mostly rural areas. Consider a person who lives in Kigali with 50 towers within a 5 km radius. This individual could regularly move within only this 5 km disk, but their CDRs would document them as using 50 towers and their mobility could be then calculated as high. Compare this person to a second person living in a rural area with only one tower in a 5 km radius of their home. Even if they move about this 5 km disk as often as our urban individual, the rural individual would only ever use this one tower and thus be classified as not moving anywhere and attain the lowest mobility rating. Thus, if not taken into account, variations in tower density create variations in mobility that do not actually exist. In other words, mobility measures that rely on cellular towers are not standardized; such measures are not comparable across areas with different tower densities. Furthermore, a single call can be transferred from one tower to another (call balancing), without any movement of the caller, due to network capacity and signal strength. In this case, mobility would be recorded when it did not in fact occur. This situation of transfer between towers happens in areas with higher tower density. These issues with tower density are further exacerbated by the fact that cellular towers are placed more often in urban areas with high population density, politically important areas, such as capital cities, or wealthy areas with higher mobile phone penetration. In short, because tower density is confounded with social, economic, political, and demographic characteristics of context, existing mobility measures that rely on tower density are confounded with these contextual factors as well.

A second, and related, concern is that the placement of cellular towers varies with time. In many countries, where the mobile infrastructure has not yet reached saturation, new cellular towers are built every year to accommodate increasing numbers of users. For example, the total 269 towers in Rwanda existed in various time periods from June 2005 to January 2009 and Fig C in S1 Supporting Information documents the growth in the number of Rwandan callers from 190 thousands in June 2005 to more than 1 million in December 2008. Towers are added in the proximity of other towers, but also in regions without previous cellular coverage—see Figs D and E in S1 Supporting Information, while others are taken off the grid. This creates a situation where the spatial density of cellular towers, which is already a problem for existing CDR-based mobility measures, is time-varying. In other words, there is temporal variance in the spatial variance of cellular towers. Because existing measures use towers as their spatial reference points, this causes a situation of spatial and temporal bias in these measures.

In addition to the problem that existing measures are confounded with tower density, they are also inherently confounded with call frequency. The more often a person calls, the more towers at which they will be registered. A person who uses their phone frequently will likely have a different mobility rating, compared to a person with the same spatiotemporal trajectory but lower calling frequency. This problem is particularly acute given that call frequency is selective of men and wealthier people [21]. Confounding with call frequency is essentially a missing data problem and creates inconsistencies between individuals. An analogous missing data problem is with areas that have no cellular towers. CDRs do not account for movement of people in areas with no tower coverage. Thus this zero tower issue is also a missing data problem, but creates inconsistencies between areas.

The temporal and spatial sparsity of CDRs [18] that affects the call frequency and zero towers problems becomes apparent in the spatiotemporal trajectory of the highest RoG caller 𝓟. This person made only two calls near two distant Rwandan borders. Given the time elapsed between the two calls and Rwanda’s transportation infrastructure, it is very unlikely that 𝓟 traveled by air between the two locations. Therefore 𝓟 was most likely present in several other locations in Rwanda, somewhere along the way between the two towers that handled their two calls. This leads to underestimates of the values of NTU, DT-SL and MDT. Even more serious is the fact that locations with cellular coverage that were visited but not represented in the CDRs can have a significant effect in the determination of the location of the center of mass, which subsequently translates into biased values of RoG.

A fourth problem is that the existing measures of mobility are fundamentally based on implicit, yet unrealistic assumptions about the nature of human movement. Their definitions involve measurement of distances in straight lines between cellular towers. In fact, humans almost never travel in straight lines and outside of air travel (which we discuss in S1 Supporting Information, Section SI4) they do not usually appear in one place, then disappear and appear again in another distant place. For example, with caller 𝓟, who was registered as being present in one side of Rwanda, then again in another side of the country, it is likely that he/she traveled longer distances on roads between these points. The straight line distance between the towers used is 236.8 km, but the quickest road route between the same locations measures 432.1 km—an increase of 82%. This clearly causes underestimates in the values of DT-SL and MDT and varying bias in RoG.

The fifth problem is that it is not entirely clear which aspect(s) of mobility most of these measures capture. Due to varying density of cellular towers, the NTU measure does not capture spatial range. However, because it counts unique towers, it also does not assess frequency of movement. The DT-SL measure captures both frequency of movement and spatial range. The MDT measure, because it incorporates only two of the towers used, captures neither frequency nor spatial range well. The RoG measure does not capture frequency of movement. While it initially appears to capture spatial range, it does so in an inconsistent manner that is influenced by call frequency from each tower used. Take the example of caller 𝓟 (see Fig 1) and of another fictive caller, 𝓟′, who makes 1000 calls from the tower used by 𝓟 for their first call, and only one call from the tower used by 𝓟 for their second call. The center of mass of 𝓟′’s trajectory will be very close (236.5 meters away) to the location of the tower used by 𝓟 for their first call, and will be 118.1 km away from the center of mass of 𝓟’s trajectory. Thus, despite the spatial range covered by 𝓟 and 𝓟′ being exactly the same, the value of RoG for 𝓟′ will be 7.5 km which is very small compared to 118.6 km, the value of RoG of 𝓟. In summary, three of the four existing measures (NTU, MDT and RoG) do not clearly and consistently measure either of the key dimensions of mobility. Only DT-SL, which incorporates both frequency and spatial range does so. Yet even this measure suffers from the major shortcomings outlined above.

## New CDR-Based Measures of Mobility

Given these concerns about existing measures of mobility, our intent is to design new measures that: (i) are standardized and independent of mobile tower density and the social characteristics of context that influence tower density; (ii) are less dependent on users’ call frequency, movement in areas with no tower coverage, and the temporal dynamics of the underlying cellular network of towers; (iii) measure clearly defined aspects of mobility such as the frequency and spatial range of movement; and (iv) are relevant and comparable across contexts, countries, and time.

The first foundation of our measures is a system of grid cells of even size placed across a country or area of study. Using grid systems to effectively standardize measurements has a long history in the geographic sciences which we draw on. To effectively address our design priorities (i)–(iv) above, and to partially address the modifiable areal unit problem (MAUP), the grid cells must be of even size and cover the entire area under study. MAUP occurs in research that defines geographic areas within which an aggregate population is analyzed. MAUP identifies the inevitable statistical bias that occurs due to scale (i.e., different sized spatial units) and zoning (i.e., different definitions of boundaries used to define spatial units) [34]. Although there is no infallible solution for MAUP [35], research has identified several methods to compensate for most related sources of bias. One such method is to use consistently sized units of geographic analysis (such as our grid cells) which provide more consistent statistical estimates and better model fit than randomly assigned units of varying sizes. [36, 37].

The size of grid cells should be chosen in relation to context and the research questions of interest for validity and as a method to reduce MAUP bias [38]. For Rwanda we chose to work with 2040 grid cells each measuring 5 km x 5 km in order to reflect the functional radius of cellular towers in that context. Specifically, each tower has a rough hypothetical radius of 5–10 km, but this is functionally reduced by the hilly terrain of much of the country.

Of key consideration is that the size of grid cells can influence error in several ways. We believe that 5 km x 5 km is the best size for national level studies, however smaller cells are feasible and likely more appropriate for smaller area studies in places with dense tower networks. Irrespective of their size, evenly sized grid cells are essential. Additional explanations related to the practical implementation of a grid system for mobility measurement, including how the grid is placed on a map and the size of grid cells, are discussed in S1 Supporting Information, Section SI1.

As shown in Figs 1 and B in S1 Supporting Information, some grid cells have a cellular tower in them, some do not, and some have multiple cellular towers. We refer to a grid cell with at least one active tower as a site. With the grid system, if an example person, 𝓡, calls from a cellular tower, we register them as being located at the centroid of the corresponding site (grid cell). Movement is then calculated only when 𝓡 moves from one site to another. If 𝓡 calls again from another tower in the same site, then they are registered in the same site, and thus have not moved. But if the next call 𝓡 makes is handled by a tower in a different site, then they have moved. Our methodology entirely disposes of cellular towers and instead replaces them with the sites they belong to. By doing so, the problem of spatial variation in tower density is eliminated because grid cells are of even size and non-overlapping. Another method for parceling a study area is to use a Voronoi tessellation, which creates cells that each encompass an area that is closer to that tower than any other. This method, which is commonly used in the CDR literature (see, for example, [22, 23, 31]), entirely covers a study area, but the differential sized cells create differential measurements of mobility depending on tower density and are subject to MAUP. In other words, using a Voronoi tessellation creates spatial variation and bias in mobility measurement. This also partially ameliorates the problem with fictitious movement being recorded when a call is transferred from one tower to another. If the two towers between which a call is transferred are located in the same site, then no movement is recorded. In some cases, the towers will be located in different sites, and movement will be recorded when it did not occur.

By replacing cellular towers with sites, the adverse effect of the temporal variability of the spatial extent of cellular towers coverage is also significantly diminished. In a given time period, a tower is active if it handled at least one cellular communication during that period. Otherwise a tower is inactive and does not contribute to the creation of a site. Fig D in S1 Supporting Information shows that, in the Rwandan data, the month to month increase in the number of sites is much smaller than the month to month increase in the number of active towers. Spatiotemporal trajectories constructed with respect to sites instead of cellular towers will have less temporally induced bias as the set of sites will always change less than or equal to the set of active cellular towers during any time period.

The second foundation of our measures is a set of realistic assumptions about how humans travel: they most often use roads, will take the quickest, most accessible road route from one place to another, and the speed of travel is affected by speed limits and quality of road surfaces. Again, using road routes, instead of straight-line distances, has historical precedent in geography and other social sciences [39–41]. With these assumptions, we use publicly available GIS data on road systems to create routes of travel from one place to another that are not straight lines—see Section SI1 in S1 Supporting Information. It is then possible to calculate an assumed route of travel between any two points in a country, where the assumed route has the shortest possible travel time compared to all other routes. Because all our measures are based on a grid system, we create assumed routes of travel that begin at the centroid of a site from which a person placed a call, take the shortest distance route to the nearest road from the site’s centroid, travel the quickest route of travel to the site in which their next call was placed, and end at the centroid of that site.

The third foundation of our measures is that humans most often travel on the ground. Even if they do not make calls at every place they visit, we can assume they existed for some amount of time in every place along a road route, between two subsequent calls. This assumption partially ameliorates the confounding influence of call frequency and no available towers on mobility measurement. In the existing measures of mobility, only places where a person made calls are included in the spatiotemporal trajectories these measures are based on, thus higher call frequency inflates mobility ratings. Here, because we account for places where people made calls and places where they did not but likely existed for any amount of time, call frequency is less confounding. For spatiotemporal trajectories that involve longer trips with one call at their origin, another call at their destination and no calls in-between (see the example of caller 𝓟), the absence of in-between calls has a reduced effect on our proposed measures of mobility because we also include in the trajectory sites and grid cells located on the quickest road routes—see Fig 1.

Based on these foundations we create six new mobility measures, and divide them into three groups depending on which of the two key dimensions of mobility they capture. Group A includes measures that capture the frequency of mobility, but do not capture spatial range; group B includes measures that capture spatial range, but not frequency; and group C includes measures that capture both frequency and spatial range. There is more than one measure in groups B and C and these differ primarily by unit of measurement. The measures within groups are of course related and thus correlate strongly. Below we describe these new measures, their benefits, and their limitations. We exemplify the evaluation of our new measures with the spatiotemporal trajectory of caller 𝓟 who made two calls in October 2005, one call from site 1976 and a second call from site 360 (Fig 1).

Combinations of measures that belong to every one of these three groups are needed to identify various mobility patterns that exist in a population. For example, caller 𝓟 made only one long trip, therefore their mobility will be rated as high by measures from group B, but not by measures in groups A and C. Consider two other callers 𝓟_1_ and 𝓟_2_ that go from home to work and back for 20 days each month, but 𝓟_1_’s work is 1 km from his home and 𝓟_2_’s work is 10 km from her home. Thus they move with equal frequency in a given period of time, but distances between consecutive places in 𝓟_1_’s trajectory are shorter than those for 𝓟_2_’s trajectory. In this case, the mobility of 𝓟_1_ and 𝓟_2_ will be equal when evaluated by measures from group A, but will differ when evaluated by measures from groups B and C. The particular measure or combination of measures one uses will depend on the research question and context of each study. We advocate at least testing analyses with all six mobility measures.

### Group A: Measures of frequency of mobility

**Number of trips (NT)**. This measure is a count of the number of times a person makes a call from a different grid cell than the previous call. It is similar to existing methods of calculating trips with CDR data [28, 32], except that this new measure is based on movement between grid cells instead of towers. Specifically, [32] require a caller to remain in one place for 10 minutes in order to record that place as a “stay location” in recording trips. Other locations, where a person remains for less than 10 minutes of recorded time are considered “pass-by” locations and not used in calculating trips. Our method of mobility measurement can be adjusted in this way as desired.

If a person makes a call from one grid cell and their next call is from the same grid cell (regardless if it is from a different tower) then this is not a trip. 𝓟 made two calls from two different sites, thus the value of NT is equal to 1 (10th percentile). If 𝓟 would have made any number of subsequent calls using only the two towers from site 360, the value of NT would be unchanged. Note that this measure does not depend on how far a person travels (spatial range): 𝓟 could have called from any two of the active sites, and the value of NT would be the same. But if 𝓟 makes a call from another site, the value of NT will increase by 1. The amount of time between the calls is disregarded when calculating NT.

The limitations of NT come from the incomplete information on mobility contained in CDRs. This measure relies on a specific definition of a trip as a movement between two places where a person existed for any amount of time. The transportation literature often defines a trip as movement between two places where a person stayed for a minimum amount of time (often 5 or 10 minutes)—see, for example, [33]. Using CDRs, it is not possible to determine how long a person stayed at each place they made a phone call. This CDR-derived measure could record fewer trips if a minimum time at a destination were required or if a person does not make a call when at a particular destination before leaving for their next destination. More trips would be recorded in cases where a person makes several phone calls when traveling between an origin and destination (or makes longer calls using multiple towers), and no minimum time at a destination were required. However, this limitation is precisely what makes NT comparable across time and place. Definitions of a trip that use any more information than we do here, will necessarily be time and context specific; an intricate definition of what constitutes a meaningful trip in rural Mongolia will certainly be different from what constitutes a trip in New York City. Thus, the limited information that NT uses is both a detraction and a benefit.

### Group B: Measures of spatial range of mobility

The next two measures represent the number of places that a person visited. Just as with trips, a careful definition of what constitutes a place is required for consistency and comparability across geographic contexts and time. Both group B measures require an assumption that all places in which a person exists for any amount of time could be important. Some of these places are marked by a person making a call. However, there are other places that a person travels through on a road route in which they did not make a call. The logic behind this assumption is fundamentally that of a missing data problem: we do not know how long a person stayed in each place, how important was each place to a particular person, or if places where they made calls were more or less important than other places they traveled through. Consequently, these measures assume all places along a person’s road route are of equal importance and counts them all.

To calculate the group B measures, we take every pair of sites that are consecutive in a spatiotemporal trajectory *M* and identify the grid cells that belong to the quickest road route between the two sites. We form the set of all the grid cells 𝓥(*M*) on these quickest road routes which also include their start and end cells, the sites from which calls were made. A grid cell appears only once in 𝓥(*M*). The elements of 𝓥(*M*) are called visited grid cells. The visited grid cells that have cellular towers in them are called visited sites.

**Grid cells visited (GCV-R)**. This measure is given by the number of visited grid cells. The GCV-R measure for 𝓟 is equal to 93 (98th percentile) because there are 93 visited grid cells between sites 1976 and 360. This measure relies on the assumptions that a person must have existed on the ground in places between subsequent calls and that, without further information, all places a person might have visited are equally important.

**Sites visited (SV-R)**. This measure is the ratio between the number of visited sites and the total number of sites in the reference time period of the trajectory *M*. As discussed in S1 Supporting Information, Section SI2, the number of sites could change from a reference time period to another as cellular towers are installed or decommissioned. Thus adjusting for the time varying number of sites is required to define a measure whose values are consistent across reference time periods. For example, 19 out of the 93 visited grid cells between sites 1976 and 360 were sites in October 2005. Since the total number of sites in October 2005 is 53, the measure SV-R is 19/53 = 0.358 (98th percentile) for 𝓟. The definition of the SV-R measure is based on the assumption that there is something important about where a cellular tower is placed, either high population density, high through-traffic, near an important area, at a cross-roads, etc. The reason that cellular towers are located in certain areas might differ between contexts and across time, but what does not differ is that there is likely a reason for cellular tower placement. We use this particular assumption because it assumes the least possible in order to define a place and is therefore the most comparable across contexts and time.

These two measures take into account the spatial range of a person’s mobility: the further each trip, the larger the number of sites and grid cells visited. But the frequency of movement is not captured by these measures. If 𝓟 would make a third call from site 1976, a fourth call from site 360, a fifth call from site 1976, and so on, the values of GCV-R and SV-R will remain the same.

### Group C: Measures of spatial range and frequency of mobility

The final three measures of mobility calculate the sum of distances between sites where consecutive communication episodes occurred. They differ only in terms of the type of units of distance used. These distances are related to the quickest of all possible road routes between two sites that are consecutive in a spatiotemporal trajectory.

**Distance traveled (DT-R)**. The distance metric for this measure is the length of the quickest road route. There are two key differences between DT-R and the existing measure DT-SL: (i) DT-SL involves movement between cellular towers, while DT-R involves movement between sites; and (ii) DT-SL is the sum of straight line distances, while DT-R is the sum of distances via road travel. If two consecutive calls were made using two towers that belong to the same site, DT-SL will record the straight line distance between the two towers while DT-R will not record any movement. On the other hand, DT-SL will underestimate distances between two points since straight line distances are almost always (if not always) smaller than distances via roads. The DT-R measure of 𝓟 is 432.1 km (38th percentile) since this is the length of the quickest road route between the two sites from which 𝓟 called.

**Time traveled (TT-R)**. The distance metric for this measure is the estimated travel time on the quickest road route. Travel time can be estimated in several ways. Speed limits can be used where available. If speed limit information is not available or the quality of roads is such that speed limits cannot be met, then average travel speeds must estimated for each type of road—see S1 Supporting Information, Section SI1.1. The TT-R of 𝓟 is 6 hours and 4 minutes (33rd percentile) since this is the smallest estimated travel time via roads between the two sites from which calls were made.

**Grid cells traveled (GCT-R)**. The distance metric for this measure is the number of grid cells intersected by the quickest road route. The start site of a route is counted, but the end site is not counted. Sites that are both the end of one route and the beginning of another are not counted twice. The GCT-R of 𝓟 is 92 (42nd percentile) since there are 93 grid cells on the quickest road route between the two sites from which 𝓟 called, including the start and end sites.

These three measures all incorporate the frequency and the spatial range of a person’s mobility. The more times a person moves, the higher will be their distance, time, and grid cells traveled. The further each trip, the higher will be these measures as well. When jointly employed, these three distance metrics are useful in identifying various patterns of mobility. For example, the mobility of two individuals that travel the same distance but use different types of roads (e.g., highways vs. minor country roads) will be rated the same by DT-R, but will differ with respect to TT-R. The distance metric for GCT-R is less dependent on particular shapes of the roads or their quality which might help when comparing mobility for spatial trajectories recorded in distant regions or countries.

## Assessment of the Proposed Measures of Mobility

The assessment of CDR mobility measures is limited by the reality that there currently exists no standard measure of mobility, or no gold standard to which we can compare new measures. In this regard, the most important assessment tool available is face validity. In other words, the best assessment tool is a careful discussion of which measures make sense and if they actually measure what we think they should be measuring. Part of this face validity discussion is above in the description of the measures, dimensions of mobility, and assumptions required for each measure.

We undertake additional assessment of our six new measures against the existing measures of mobility and against each other by estimating longitudinal pairwise correlations based on the spatiotemporal trajectories of callers for each of 44 months of Rwandan CDRs. Results, figures and a discussion are presented in S1 Supporting Information, Section SI5. All longitudinal associations are positive with values from medium to high, and are very stable through time. Measures within groups have the strongest associations, as we would expect. But other, less intuitive high associations emerge, especially between certain existing and new measures. In particular, the DT-SL measure, which is conceptually consistent with both key dimensions of mobility, has the strongest associations with the measures in group B (spatial range), and only the second strongest associations with the measures in group C (spatial range and frequency). While this is somewhat surprising, it also emphasizes the fundamental differences in the way DT-SL is defined as opposed to our new measures, especially DT-R.

In addition to face validity and correlations, it is important to assess which groups of measures and which measures within each group should be used for studies of population mobility. The six measures we introduce offer multiple choices of combinations which could be selected based on particular research questions and contexts. We also argue that all our six measures are needed in principled, thorough population mobility studies. Despite having common characteristics in terms of the two key dimensions of mobility we discussed, each measure captures a slightly different aspect of mobility, and is thus relevant alone as well as jointly with the other five measures. To demonstrate this, we used our six measures to define categories of callers with different mobility profiles for four of the 44 months of data (June 2005 to January 2009)—see S1 Supporting Information, Section SI6. A monthly spatiotemporal trajectory of a caller was classified as having high or low mobility with respect to a measure if the value of their mobility measure was above or below the median of observed values during that month. For each month, with six separate measures this leads to 64 categories. Tables A, B, C and D in S1 Supporting Information show that at least 11 of the 64 categories contain at least 1.0% of the callers in each of the four months we examined. This suggests that there are many distinct mobility types in this population that can only be identified by using all six measures in combination. Overlooking any one measure would lead to conflating segments of the population with distinct mobility profiles.

Further, we find several notable patterns with these tables. Two profiles which rate mobility as low or high for all six measures are the largest ones in all four months, and comprise about 30% of the monthly callers. The third and fourth largest segments rate mobility as low or high for groups A and C, and as the opposite for group B. These segments comprise about 7% of the monthly callers, and show the relevance of capturing frequency of mobility (groups A and C) versus spatial range (group B). Another notable result is the relatively common mobility profiles that rank high on one spatial range measure, low on the other spatial range measure, and high or low on all other measures. These two groups comprise about 5% of the population and indicate people who likely travel often and far, but mostly in areas with few cellular towers (thus a high GCV-R but low SV-R). In contrast, there are people who travel seldom and short distances, but in areas with many cellular towers (thus a low GCV-R but high SV-R). Again, assessing all six mobility measures for each individual is necessary to identify particular mobility types in a population.

The mobility measure time traveled (TT-R) has an additional important use. As we show in S1 Supporting Information, Section SI7, the values of this measure can be used to identify and possibly filter out spatiotemporal trajectories affected by errors in cellular services provider’s databases, by intruders who gain unauthorized access to mobile phones and use them to communicate at the same time as the actual owners or by robot-based callers [42]. The identification of such unusual trajectories is not possible with the existing measures of mobility.

## Discussion

Our mobility measures are designed to be applicable to any research setting, from wealthy countries with well-developed mobile phone and transportation infrastructure, to poorer countries that are yet developing transportation and communication networks. They constitute an important advance in the social scientific study of mobility which could lead to improved understanding of human health and well-being and macro-economic, social, political, and demographic dynamics. Being almost entirely spatially derived, and using CDRs enhanced with GIS data, these new measures circumvent many of the problems inherent in existing mobility measures and are independent of cellular tower density and the social, political, economic, or demographic characteristics that influence tower density. They are thus relevant and comparable in different contexts. Another key goal with this paper is to stimulate discussions on mobility measures using CDRs, and to promote social science research on the causes and consequences of human mobility. In this regard, we herewith discuss some of the many ways in which these new CDR-based measures of mobility can be used to enhance and expand our understanding of human well-being and social organization.

This comparability is achieved by keeping our general approach relatively simple and through a set of assumptions about how humans travel. While we advocate for simplicity and parsimony in using these measures, our calculations and assumptions can be adjusted to make the measures more relevant for the specific context of any given study. For example, our approach assumes that people most often travel on land and via roads (instead of by air), and in cars or buses (instead of by foot or via public transportation). In many countries and cities, air travel and public transportation are commonly used, and our system will require adjustment to address these possibilities. We provide some ideas for how these adjustments could be made in S1 Supporting Information, Section SI4, but note that further effort and testing will undoubtedly improve on our preliminary suggestions here.

Our mobility measures quantify the movement of callers across shorter or longer time periods, e.g. several hours, one day or one month. Conceptually, these measures differ from approaches that measure mobility by (i) identifying key locations of each caller (e.g., home, work or other), (ii) identifying trips between these locations, and (iii) counting the number of trips between any two locations across all callers, and recording these counts in origin-destination (OD) matrices [28, 32]. OD-matrices capture ecological (group-level) movement between locations, while our mobility measures capture the individual-level movement of each caller across all the locations they visited in a fixed time interval. Once recorded in an OD-matrix, the link between the trips made by the same caller is lost. In our approach for measuring mobility, this link is preserved.

First, these new measures can replace older measures, often based on sample surveys, to improve understanding of existing mobility related questions. The benefit here is clear, given that CDR-based measures can significantly increase the accuracy, detail, and time period over which mobility can be recorded. They are also much less costly to obtain than detailed survey measurements. CDRs can be collected and measures of mobility calculated for respondents who participate in sample surveys, giving the researcher not only immense detail about respondent mobility, but also the opportunity to compare it with survey records of other characteristics and behaviors.

Second, these new measures open up entirely new avenues of research. Because CDRs can cover millions of people, it is possible to calculate population-level mobility measures. For example, one can calculate a measure of general mobility for a city, state, province, or region. This could then be compared to individual level behaviors and outcomes to investigate questions such as how population mobility influences individual migration, tuberculosis infection, or women‘s labor force participation. Population level mobility can also be related to population-level characteristics, such as HIV prevalence rates, birth rates, social norms, economic well-being, or political participation. With sample surveys, it has never before been possible to calculate population level characteristics, thus CDR-based measures, if appropriately calculated to be independent of tower density and the related contextual characteristics, create new and possibly groundbreaking opportunities for social science.

Third, CDR-derived population level measures mobility can be used to identify and study emergency events, such as natural disasters and armed conflict. For example, theory and evidence predict that people will change their mobility patterns during and after an earthquake or a large bomb blast [25, 43]. With access to real time data, it would then be possible to pinpoint an earthquake or bomb blast in real time, even in remote areas with poor communication and transportation linkages. Given that the time to humanitarian response significantly influences the magnitude and time of the post-event disaster period, real-time identification of hazardous events could ultimately lead to decreasing the human toll of disasters. By employing one of the mobility measures proposed in this article (number of trips, NT), our group has recently made significant advances in the identification of changes in human population behavior associated with earthquakes, floods, violence against civilians and protests [44].

While CDR-based measures can create immense new opportunities for understanding human mobility, there are several limitations of which researchers must be aware. As with all organic big data [45], selection is a major concern. For mobile phone data, mobile phone users are included in the data set and non-users are excluded. Research suggests that users are more likely to be male, educated, and live in urban areas [21, 23]. Alternately, research has also shown that there are an estimated 90.2 mobile phones per 100 people in poorer countries [20]. Considering that mobile phone penetration statistics are largely analogous to response rates in surveys, we can say that CDR based data essentially have a 90.2% response rate in poorer countries, which is generally considered good if not excellent, regardless of selection.

Another key limitation to the use of CDR-based mobility measures is the inherent error. The primary problem is that although mobile calls are recorded as occurring at a cellular tower, the person making the call is rarely at that tower. Instead they are likely to be within 5 or 10 km from the tower, depending on the type of antenna used in the tower and topography. Other limitations to CDR-based mobility measures are that infrequent mobile phone use and transfers of calls between towers (when the caller does not actually move) can create error. Our grid cell system partially, but not completely, ameliorates these possible sources of error. Regardless, we argue that the benefits of CDR-based mobility measures vastly outweigh the detractions, especially when compared to the alternative of survey-based measures with inherent error due to human difficulties in recalling location, time, and movement accurately and the inability to measure population level mobility.

## Supporting Information

S1 Supporting Information Supplementary text and figures. (PDF)
