# Supplementary material for: Measures of Human Mobility Using Mobile Phone Records Enhanced with GIS Data
Source: PLoS One. 2015 Jul 20;10(7):e0133630. doi: 10.1371/journal.pone.0133630 (PMC4507852; doi:10.1371/journal.pone.0133630)
Supplement: S1 Supporting Information — (PDF) [file pone.0133630.s001.pdf]

SUPPORTING INFORMATION FOR "MEASURES OF HUMAN MOBILITY USING MOBILE PHONE RECORDS ENHANCED WITH GIS DATA"

N. WILLIAMS, T. THOMAS, M. DUNBAR, N. EAGLE, A. DOBRA

Contents

List of Figures 1
SI1: The Road Network and the Grid Cell System 2
SI1.1: The Road Network System 2
SI1.2: The Grid Cell System 5
SI2: The Temporal Dynamics of the Cellular Network 6
SI3: Measures of Mobility 7
SI3.1: Existing Measures of Mobility 8
SI3.2: New Measures of Mobility 10
SI4: Addressing the Possibility of Air Travel for the Proposed Mobility Measures 11
SI5: Longitudinal Pairwise Associations of Measures of Mobility 12
SI6: Categories of callers defined by their mobility with respect to the measures in Groups A, B and C 22
SI7: Checking the Quality of CDRs with the Measure of Mobility Time Traveled (TT-R) 27
References 29

List of Figures

A Map of Rwanda showing the position of the 269 cellular towers (red) and the structure of the network of roads (trunk, primary, secondary and tertiary) used for our mobility measures (gray). Roads that are also segments on quickest routes are shown in blue. 3
B Map of Rwanda showing the position of the cellular towers (red) with respect to the 2040 5 km x 5 km grid cells. Rwanda's boundary is shown in blue. 4
C Number of callers during each month between June 2005 and January 2009. 7
D Number of active cellular towers (blue) and sites (green) during each month between June 2005 and January 2009. 8

E Location of sites during four months: December 2005, 2006, 2007 and 2008. In each of the four panels, red denotes grid cells that were not sites the year before. All the other sites are shown in blue. This plot reveals that a large number of cellular towers were installed between December 2007 and December 2008 which significantly increased the spatial coverage of the wireless services provider that provided the Rwandan CDRs. 9

F Estimated correlations for each of the 44 months of Rwandan CDRs between the existing measure of mobility number of towers used (NTU) and the six measures from Groups A (blue), B (shades of green) and C (shades of red). 13

G Estimated correlations for each of the 44 months of Rwandan CDRs between the existing measure of mobility distance traveled (DT-SL) and the six measures from Groups A (blue), B (shades of green) and C (shades of red). 14

H Estimated correlations for each of the 44 months of Rwandan CDRs between the existing measure of mobility maximum distance traveled (MDT) and the six measures from Groups A (blue), B (shades of green) and C (shades of red). 15

I Estimated correlations for each of the 44 months of Rwandan CDRs between the existing measure of mobility radius of gyration (RoG) and the six measures from Groups A (blue), B (shades of green) and C (shades of red). 16

J Estimated correlations for each of the 44 months of Rwandan CDRs between the new measure of mobility number of trips (NT) which defines group A, and the five measures from Groups B (shades of green) and C (shades of red). 17

K Estimated correlations for each of the 44 months of Rwandan CDRs between the new measure of mobility grid cells visited (GCV-R) from group B, and the other measure of mobility from group B (SV-R, green), as well as the four measures from Groups A (blue) and C (shades of red). 18

L Estimated correlations for each of the 44 months of Rwandan CDRs between the new measure of mobility sites visited (SV-R) from group B, and the other measure of mobility from group B (GCV-R, green), as well as the four measures from Groups A (blue) and C (shades of red). 19

M Estimated correlations for each of the 44 months of Rwandan CDRs between the new measure of mobility distance traveled (DT-R) from group C, and the other measures of mobility from group C (TT-R and GCT-R, shades of red), as well as the three measures from Groups A (blue) and B (shades of green). 20

N Estimated correlations for each of the 44 months of Rwandan CDRs between the new measure of mobility time traveled (TT-R) from group C, and the other measures of mobility from group C (DT-R and GCT-R, shades of red), as well as the three measures from Groups A (blue) and B (shades of green). 21

O Estimated correlations for each of the 44 months of Rwandan CDRs between the new measure of mobility grid cells traveled (GCT-R) from group C, and the other

measures of mobility from group C (DT-R and TT-R, shades of red), as well as the three measures from Groups A (blue) and B (shades of green). 22

SI1: The Road Network and the Grid Cell System

Two of our methodological developments that are based on Geographic Information Systems (GIS) are the road network system from Rwanda and the grid cell system which divides a spatial bounding box for Rwanda's boundary into 2040 5 km x 5 km cells. With any GIS, the choice of an appropriate coordinate system, determined by the geographic location and scale of analysis, is required to assure accurate measures of distance and area. Our road network and grid cell system are based in the Universal Transverse Mercator (UTM) zone 36S coordinate system, using the WGS84 datum[1]. The GIS data that is necessary to replicate the results presented in the paper are available for download from:

`https://github.com/adobra/RwandaMobility.git`

Figures A and B display the locations of the 269 cellular towers that appear in the Rwandan CDR data with respect to the road network and the grid cell system, respectively. The grid cells that contain at least one tower are called sites. Only 155 out of the 2040 grid cells are sites. Four sites in the Kigali area contain the largest number of cellular towers: 41, 22, 6 and 5, respectively. Seven sites contain four towers, four sites contain three towers, 14 sites contain two towers and the other sites contain only one tower. These counts represent the towers that belong to a site between June 1, 2005 and January 31, 2009. In any period of time between these dates, all, some or none of the towers that belong to a site are actually active (i.e. handle cellular communications). As such, the number of sites (i.e., grid cells that contain at least one active tower) in a time period might be smaller than 155 – see Section SI2.

SI1.1: The Road Network System. A key feature of our methodology is utilizing road networks to estimate travel distances and travel times between locations. We evaluated several publicly available road network datasets for Rwanda, and determined that the crowd sourced OpenStreetMap[2] (OSM, henceforth) currently provides the most detailed and up-to-date road network information. OSM also provides data on the quality of the roads with respect to which roads can be categorized in the following hierarchy: trunk roads, primary roads, secondary roads and tertiary roads. However, OSM is volunteer created and has some discrepancies in the interpretation and classification of certain roads (e.g., trunk vs. unclassified). Since OSMs road network does not contain speed limit or road conditions, we must estimate the likely average speed with which individuals travel on roads of different types based on assumed road conditions and probable posted speed limits. Trunk roads, the top category of the hierarchy, are likely to be paved and allow higher speed limits, so we assume that someone would travel with an average of 120 km/h on these roads. On primary roads, which are probably paved but with slower traffic, we assume that individuals travel with an average of 60 km/h. Secondary roads are most

[1]Such types of linear and areal measurements cannot be performed in a GIS using an unprojected or spherical coordinate system, such as degrees latitude and longitude, which measure angles rather than surface distance.

[2]`http://www.openstreetmap.org/`

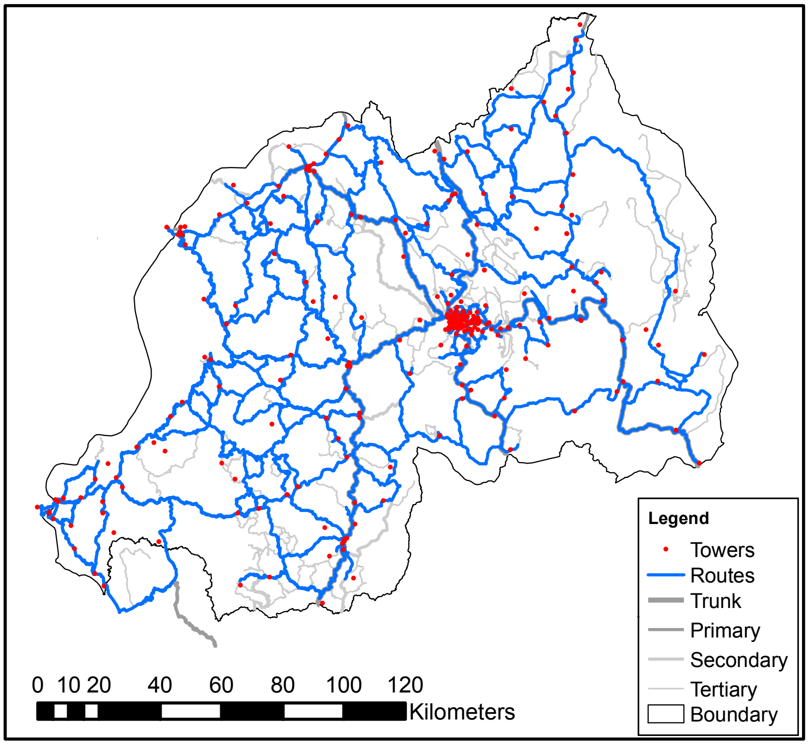

Figure A. Map of Rwanda showing the position of the 269 cellular towers (red) and the structure of the network of roads (trunk, primary, secondary and tertiary) used for our mobility measures (gray). Roads that are also segments on quickest routes are shown in blue.

likely a mix of paved and unpaved surfaces, thus we assume that individuals travel with an average speed of 45 km/h on these roads. Tertiary roads are likely to be unpaved and are probably more difficult to travel at higher speeds for various reasons (e.g., traffic, population density, remoteness, poor conditions of the road). We assume an average travel speed of 30 km/h on tertiary roads. Note that it is the ratio between assumed speeds on different road types that is important, rather than the absolute magnitude of each speed.

One of the caveats of OSM's road network data for Rwanda is that there are some segments of the roads that did not connect with the rest. Since our methodological approach for calculating mobility measures is based on the assumption that any two locations in the country are connected by traveling along the road network, we joined those disconnected road segments with the rest of the network by changing the classification of 67 lesser category roads, most of which were labeled as unclassified or residential in the raw OSM data, to tertiary roads. With these additional roads, we created a connected road network for

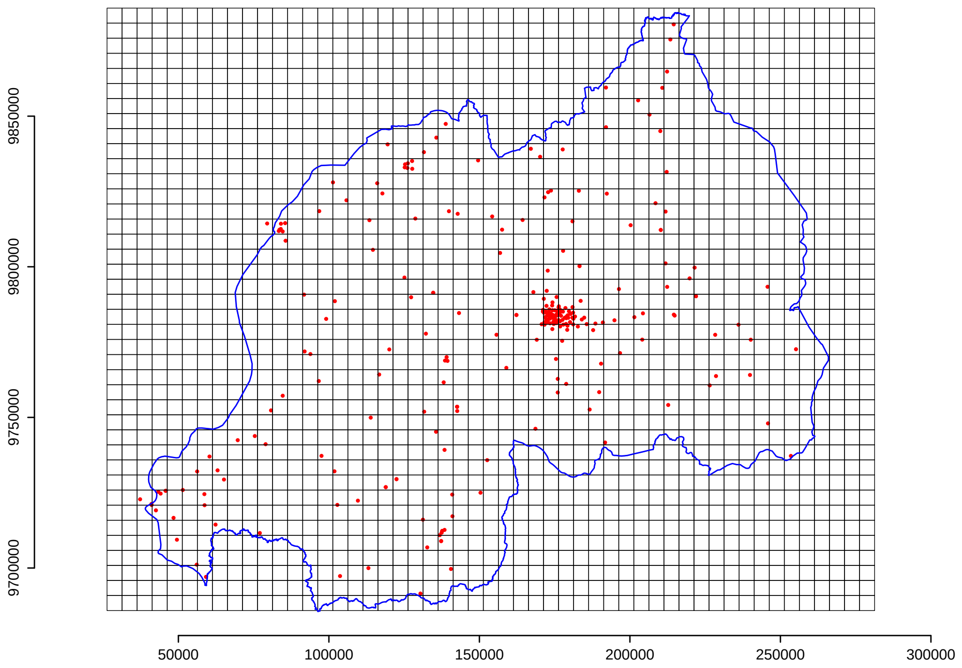

Figure B. Map of Rwanda showing the position of the cellular towers (red) with respect to the 2040 5 km x 5 km grid cells. Rwanda's boundary is shown in blue.

Rwanda which we used in the calculation of the measures of mobility in this paper – see Figure A.

After establishing a connected road network and the corresponding average travel speeds, we employed ESRIs ArcGIS[3] to determine approximate travel distances and travel times between the centroids of pairs of sites. We used the function "Closest Facility" of ArcGIS Network Analyst[4] to identify the quickest road paths between the centroids of any pair of sites and stored these $\binom{155}{2} = 23870$ routes together with their corresponding travel distances and travel times. We subsequently performed thorough quality checks to ensure the estimated travel distances and travel times are indeed realistic. The last step involved identifying the specific sites and grid cells on the quickest route between the centroids of each pair of two sites. We also stored this information in our route database.

[3]http://www.esri.com/software/arcgis
[4]http://www.esri.com/software/arcgis/extensions/networkanalyst

SI1.2: The Grid Cell System. Our approach, which involves overlaying a customized rectangular grid with square cells of equal size on the map of Rwanda, creates a systematic method of circumventing the major problem of spatial variance in cellular tower density. We replace tower locations with the centroid of the sites they belong to. Instead of measuring straight line distances from tower to tower, we measure distances between the centroids of the sites via the quickest road route which connects these centroids.

Choosing the size of the grid cells is an important decision. We are interested in country level mobility patterns and believe that 5 km x 5 km grid cells are appropriate for this purpose[5]. Based on geographical and technological considerations, we estimated catchment areas in which a user of a cellular tower is likely to be located. We estimated that the maximum signal distance for the type of towers in Rwanda is roughly 10 km. Several factors further reduce this maximum signal distance, including relative location of a user with respect to a tower, topography of the areas surrounding towers, and the decay in signal strength with increasing distances from towers. As such, we reduced the maximum user-to-tower distance to 5 km. The resulting 5 km x 5 km grid cell system is a 51 x 40 matrix (2040 grid cells) that covers 51,000 km^2 extending just outside of the border of Rwanda – see Figure B. Each grid cell is indexed by a number from 1 to 2040: grid cell 1 is located in the lower left corner and grid cell 2040 is located in the upper right corner. The indices increase first by row, then by column. Each of the 269 towers is subsequently mapped to its corresponding grid cell (site).

A possible caveat of using a grid cell system as a foundation for measuring mobility is that the spatial placement of the grid creates arbitrary boundaries that could non-systematically influence mobility measures. For example, person $\mathcal{A}$ could call from a cellular tower that is one meter away from a grid cell boundary. If $\mathcal{A}$ moves two meters to cross the grid cell boundary and makes a call from a tower in the new grid cell, $\mathcal{A}$ will be registered as moving between grid cells, even with only two meters of actual travel. On the other hand, another person $\mathcal{B}$ could call from a tower inside a grid cell, move up to $5\sqrt{2} \approx 7.07$ km without crossing a grid cell boundary, and make another call. Because $\mathcal{B}$ remained in the same grid cell, $\mathcal{B}$ would not be registered as having moved, despite traveling a lot further than $\mathcal{A}$.

There is a reasonably simple, though computationally intensive, method to account for the bias induced by the arbitrary spatial placement of the grid cells. Consider a system of 5 km x 5 km grid cells which is placed over a map of the study area, and calculate the corresponding values of the measures of mobility which are dependent on the locations of the cells. Next, move the entire grid system 1 km east over the map, and recalculate the values of the mobility measures. The grid system can be moved 25 times (by 1 km east and 1 km south each time), and the resulting 25 sets of values of the measures of mobility can be determined. These 25 sets of values can be used to produce mobility measures

[5] For other applications that involve an in-depth look at mobility within predominantly urban settings such as Rwanda's capital Kigali, the same methodology can be used with the grid cell size modified to a more appropriate scale to capture local scale mobility (e.g., 1 km x 1 km grid cells).

estimates as well as standard errors which account for the arbitrary placement of grid cell boundaries.

The raw OSM's data for Rwanda was such that 11 sites were not intersected by the Rwandan road network. To connect these sites to the road network, we moved the location of their centroids to adjacent grid cell centroids. The adjacent grid cell used was the one closest to the tower in the original cell. In a few cases this led to an overlap between two sites, one of which happened in the Kigali area which has a high tower density. We believe this is not a major problem because, as mentioned above, the coverage of a cellular tower is roughly a circular area with a 5 km radius. The distance between two centroids is exactly 5 kilometers and each of the roads that were barely missed was within 3 km of the centroid of the nearest grid cell. In all cases, we spot checked the change to make sure it was the most reasonable.

When we replace towers with grid cells, there is the possibility of increasing the error in a person's location. Notably, there is the symmetric possibility that the grid system could decrease locational error. In most cases, it is likely that the combined error (uncertainty of a person's location in relation to a tower combined with additional grid system locational error) is negligible. In the most extreme circumstance, with a 5 km x 5 km grid system, towers that broadcast to a 10 km radius, and a tower that is in a far corner of a grid cell, a person's location could be calculated as being up to 13.5 km from their actual location. Note that the majority of the error here (10 out of 13.5 km) is due to tower-location uncertainty and the minority of error (3.5 km) is due to the imposition of the grid system. This maximum possible error of 13.5 km is likely not as problematic when measuring mobility on a national scale compared to a smaller local scale. When measuring mobility on a smaller scale in areas with higher tower density, or towers that are closer to each other than 10 km, the maximum possible locational error will be less. It can be further reduced by decreasing the size of grid cells. Thus, locational error must be carefully considered when CDR-based data is used to measure mobility and further work should be done to assess the effects of selection and locational error.

SI2: The Temporal Dynamics of the Cellular Network

The network of cellular towers managed by a wireless service provider could vary significantly over months and years in terms of the total number of towers, their spatial coverage, and the number of users of the network. A cellular tower is called active in a given time period (e.g., a month or a year) if it handled at least one call during that period. We used the available Rwandan CDRs to determine the number of callers in this provider's network, and which cellular towers handled their calls every month from June 2005 to January 2009.

Figure C shows that the number of users using this cellular network increased from 190 thousands in June 2005 to 238 thousands in December 2005, 310 thousands in December 2006, 552 thousands in December 2007, and reached more than 1 million people by December 2008. Figure D reveals that the number of active towers that handled communications in this network continually increased from 73 in June 2005 to 79 in December 2005, 91 in December 2006, 136 in December 2007 and reached 246 in December 2008. During this

time the network expanded with progressively increasing tower density in more populated areas through the installation of additional towers in sites that already had towers in them, but also with progressively increasing spatial coverage through the installation of new towers in grid cells that previously did not contain any towers. The difference between these two dimensions of the dynamics of the cellular network is evident when comparing the month to month increase in the number of sites with the increase in the number of towers: increased tower density at the same sites is not captured in the number of sites during each month, but it is captured in the increase in the number of active towers. Although not obvious in this figure, there is always the possibility that a cellular network loses towers which leads to lower tower density at some sites or to sites no longer containing active towers. Increased spatial coverage is evident from the bottom panel of Figure D: there were 49 sites in June 2005, 54 sites in December 2005, 60 sites in December 2006, 76 sites in December 2007 and 143 sites in December 2008.

The expansion of the spatial coverage of this cellular network is shown in Figure E, which shows where the installation of new towers led to coverage in grid cells that previously did not have cellular service from this provider. The large expansion in spatial coverage recorded in December 2008 compared to December 2007 is especially important as it vastly improved the accessibility of mobile technology in towns and rural areas throughout Rwanda.

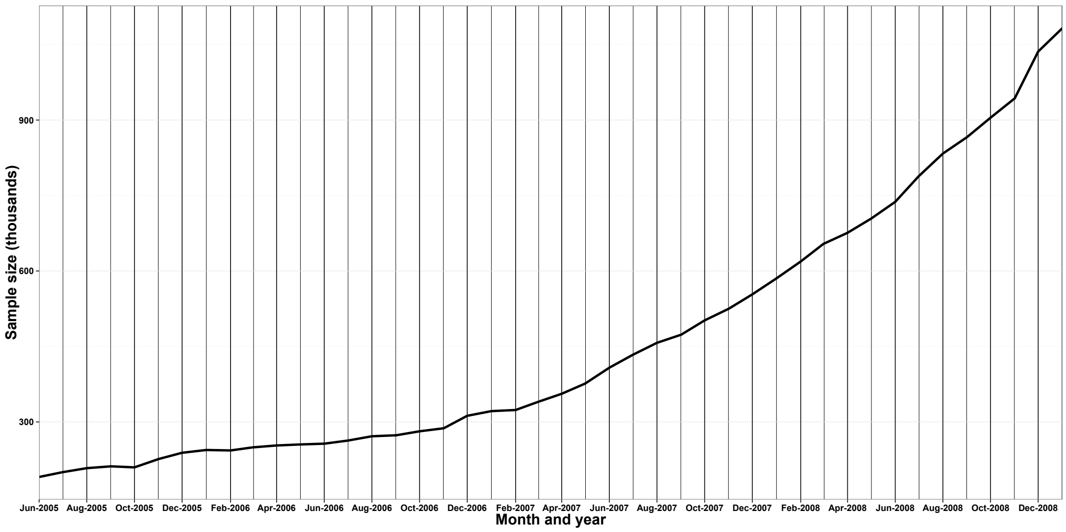

Figure C. Number of callers during each month between June 2005 and January 2009.

SI3: Measures of Mobility

Here we give formal mathematical definitions of the measures of mobility described in the main text. Consider the sequence of CDRs associated with a mobile phone in a reference

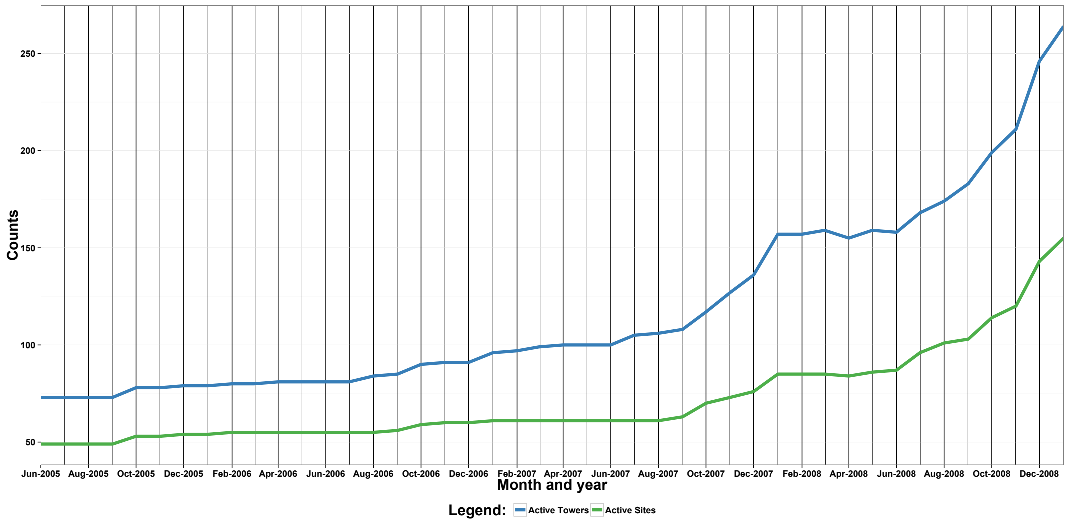

FIGURE D. Number of active cellular towers (blue) and sites (green) during each month between June 2005 and January 2009.

period of time $\mathcal{T}$ (e.g., a day, a week, a month or a year):

$$M = \{m_1, m_2, \ldots, m_n\}. \tag{S1}$$

We assume that the wireless-service provider that generated these CDRs has K active towers in the reference time period $\mathcal{T}$, and that the spatial locations l_i^{CT}, $i \in \mathcal{K} = \{1, 2, \ldots, K\}$ of these active towers are known. In (S1), $m_i \in \mathcal{K}$, $1 \leq i \leq n$, is the identifier of the cellular tower that handled the communication represented by the i-th CDR in the sequence (S1). If $i < j$ the communication represented by m_i was recorded before the communication represented by m_j. We refer to M as the spatiotemporal trajectory of the cellular phone that generated the sequence of CDRs. We remark that more than one tower might have handled the same communication (e.g., a call), but in that case several CDRs – one for each cellular tower – would have been generated.

For any pair of spatial locations l and l' identified by their latitude and longitude coordinates, we define the distance function $d_{SL}(l, l')$ which represents the straight line or "as the crow flies" distance between l and l'. We take $d_{SL}(l, l) = 0$.

SI3.1: Existing Measures of Mobility. For the spatiotemporal trajectory M from (S1), the measure of mobility called "number of towers used" (NTU) is the number of unique towers that appears in this sequence, i.e.

$$\#\left\{i : i \in \mathcal{K} \text{ such that there exists } m_j, 1 \leq j \leq n \text{ with } m_j = i\right\}.$$

Here $\#A$ denotes the number of elements in the set A. The measure of mobility called "distance traveled" (DT-SL) is the sum of straight line or "as the crow flies" distances

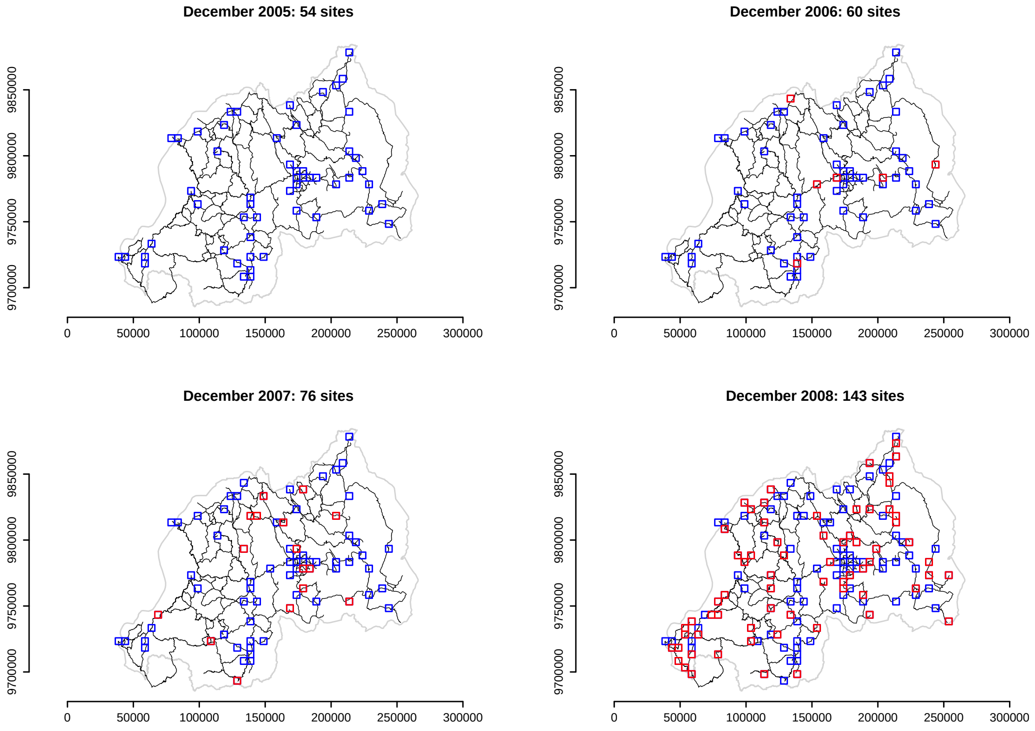

Figure E. Location of sites during four months: December 2005, 2006, 2007 and 2008. In each of the four panels, red denotes grid cells that were not sites the year before. All the other sites are shown in blue. This plot reveals that a large number of cellular towers were installed between December 2007 and December 2008 which significantly increased the spatial coverage of the wireless services provider that provided the Rwandan CDRs.

between consecutive towers from which communication occurred:

$$\sum_{j=2}^{n} d_{SL}\left(l^{CT}_{m_{j-1}}, l^{CT}_{m_j}\right).$$

The measure of mobility called "maximum distance traveled" (MDT) is the maximum straight line distance between two towers in the sequence M:

$$\max_{1\leq i<j\leq n} d_{SL}\left(l^{CT}_{m_i}, l^{CT}_{m_j}\right).$$

The measure of mobility called "radius of gyration" (RoG) is the square root of the mean of the squared straight line distances between the locations of towers in M and the center

of mass

$$\bar{l}_M = \frac{1}{n}\sum_{j=1}^{n} l^T_{m_j}, \tag{S2}$$

of the trajectory:

$$\sqrt{\frac{1}{n}\sum_{j=1}^{n} d^2_{SL}\left(l^T_{m_j}, \bar{l}_M\right)}.$$

Equation (S2) defines the center of mass as the arithmetic mean of the spatial locations of cellular towers in the trajectory M.

SI3.2: New Measures of Mobility. We assume that the region of interest was divided into non-overlapping grid cells identified by indices in $\mathcal{Q} \in \{1, 2, \ldots, Q\}$. We denote by l^{GC}_j the location of the centroid of the grid cell $j \in \mathcal{Q}$. We introduce a mapping function $q^{GC}(\cdot)$ which gives, for each cellular tower $i \in \mathcal{K}$, the grid cell $q^{GC}(i) \in \mathcal{Q}$ the tower belongs to. The sites are those grid cells that contain at least one tower:

$$\mathcal{S} = \left\{j : j \in \mathcal{Q} \text{ such that there exists } i \in \mathcal{K} \text{ with } q^{GC}(i) = j\right\}.$$

Since we assume that all the towers indexed by $\mathcal{K}$ are active in the reference time period $\mathcal{T}$, $\mathcal{S}$ represents the set of sites in $\mathcal{T}$.

We transform the spatiotemporal trajectory M from (S1) into the corresponding time ordered sequence of sites to which the active towers that appear in M belong to:

$$M^{GC} = \{g_1, g_2, \ldots, g_n\}, \tag{S3}$$

where $g_i = q^{GC}(m_i) \in \mathcal{S}$.

The measure of mobility called "number of trips" (NT) is a count of the number of times a person communicates from a different grid cell than their previous communication:

$$\#\left\{i : i \in \{1, 2, \ldots, n-1\} \text{ such that } g_i \neq g_{i+1}\right\}.$$

The other five measures of mobility introduced in the main text are constructed with respect to a road network that connects any two locations l and l' in the region of interest. If a location l is not on the road network, we assume its spatial location is replaced by its projection (the location at the smallest straight line distance) on the road network. With this convention, we assume that any two locations l and l' are connected by at least one continuous subset of locations called road route on the road network. We assume that these road routes do not contain loops. From all the road routes that connect l and l', we choose the route $\mathcal{P}(l, l')$ that is quickest, i.e. the route with the smallest estimated travel time. Each location on the path $\mathcal{P}(l, l')$ must belong to exactly one grid cell. We denote by $\mathcal{R}(l, l')$ the subset of $\mathcal{Q}$ that comprises all the grid cells intersected by the road route $\mathcal{P}(l, l')$. We define the following distance functions:

(1) $d_{RD}(l, l')$ represents the length of $\mathcal{P}(l, l')$.
(2) $d_{TT}(l, l')$ represents the estimated travel time between l and l' on $\mathcal{P}(l, l')$.
(3) $d_{GC}(l, l')$ represents the number of grid cells in $\mathcal{R}(l, l')$.

We take $d_{GC}(l,l) = d_{RD}(l,l) = d_{TT}(l,l) = 0$.

The set of visited grid cells associated with the spatiotemporal trajectory M^{GC} from (S3) are those grid cells on the fastest road routes that connect consecutive sites in M^{GC}:

$$\mathcal{V}\left(M^{GC}\right) = \bigcup_{j=2}^{n} \mathcal{P}\left(l_{g_{j-1}}^{GC}, l_{g_j}^{GC}\right).$$

The measure of mobility called "grid cells visited" (GCV-R) is given by the number of visited grid cells $\#\mathcal{V}\left(M^{GC}\right)$. The visited grid cells that are also sites are called visited sites. The measure of mobility called "sites visited" (SV-R) is given by the ratio between the number of visited sites and the total number of sites in the reference time period $\mathcal{T}$:

$$\#\left(\mathcal{V}\left(M^{GC}\right) \cap \mathcal{S}\right) / \#\mathcal{S}.$$

The measure of mobility called "distance traveled" (DT-R) is the sum of the lengths of the quickest road routes between consecutive sites from which communication has occurred:

$$\sum_{j=2}^{n} d_{RD}\left(l_{g_{j-1}}^{GC}, l_{g_j}^{GC}\right).$$

The measure of mobility called "time traveled" (TT-R) is the sum of the estimated travel times on the quickest road routes between consecutive sites from which communication has occurred:

$$\sum_{j=2}^{n} d_{TT}\left(l_{g_{j-1}}^{GC}, l_{g_j}^{GC}\right).$$

The measure of mobility called "grid cells traveled" (GCT-R) is the number of grid cells intersected by the quickest road routes between consecutive sites from which communication has occurred:

$$\sum_{j=2}^{n} d_{GC}\left(l_{g_{j-1}}^{GC}, l_{g_j}^{GC}\right).$$

SI4: Addressing the Possibility of Air Travel for the Proposed Mobility Measures

Our proposed system of calculating mobility measures is entirely based on the assumption that most people travel via land and on roads. Our empirical work employs CDRs from Rwanda which is a small country of approximately 23,338 km^2 — about the size of the U.S. state of Massachusetts. Rwanda generally ranks low on indices of human development, with an estimated 57% of the population poor and 38% extremely poor in 2006. The population density is amongst the highest in Africa, at 347 people per square km and most Rwandans (87%) live in rural areas. Despite the rather poor and rural conditions of the population and contentious history, the Rwandan economy, road networks, and living conditions of the people have improved throughout the 2000s.

As such, the assumption that most travel occurs by roads is quite reasonable for a country such as Rwanda. However, in larger and/or wealthier countries a significant proportion of

travel occurs by air instead of roads. Several adjustments must be made to our methods to account for air travel. The first step in making these adjustments is to identify when a person most probably traveled via air rather than roads. To this end, we must make explicit use of the dates and the times of generation of each CDR. If the time period between two consecutive CDRs of a person $\mathcal{A}$ associated with different grid cells G_1 and G_2 is shorter than the shortest possible travel time via roads between these grid cells, we can assume that $\mathcal{A}$ traveled via air between G_1 and G_2. As with any assumption, this inherently includes some error. This method will correctly identify all air travel for people who make calls soon before taking off and soon after landing. However, if a person does not make calls for some time before and after flying, then their travel will not be identified as such. There will be more error in this manner for flights that cover shorter distances than for longer distance flights.

Once air travel movement has been identified for pairs of consecutive CDRs, four of the six proposed mobility measures could be calculated differently. Specifically, for such pairs, sites visited (SV-R) can include only the grid cells in which $\mathcal{A}$ made consecutive calls and no other sites (grid cells with active mobile towers) on a route in between. Grid cells visited (GCV-R) can be the number of sites visited and no other grid cells on a route in between. Time traveled (TT-R) can be the difference between the times of the generation of the two consecutive CDRs. Alternately, the time that flights take between the two closest airports can be used. Distance traveled (DT-R) can be calculated as the straight line distance between the centroids of the two sites from which calls were made. However, to maintain consistency with segments of movement that most likely occurred by roads, it might be desirable to calculate all air travel based measures as if they were undertaken via road. In either case, the number of trips (NT) requires no adjustment for air travel movement.

SI5: Longitudinal Pairwise Associations of Measures of Mobility. We investigate the relationships between the existing measures of mobility (NTU, DT-SL, MDT and RoG) and the proposed measures (NT – group A; GCV-R and SV-R – group B; DT-R, TT-R and GCT-R – group C). For each of the 44 months between June 1, 2005 and January 31, 2009, we employ the spatiotemporal trajectories of callers during that month to estimate pairwise correlations between these 10 measures of mobility. We make use of the semi-parametric Bayesian Gaussian copula estimation method of [1] which produces estimates of the correlation matrix of the Gaussian copula via a Markov chain Monte Carlo (MCMC) algorithm. For each of the 44 months, we employed this MCMC algorithm to draw 10000 samples from the posterior distribution of the 10 dimensional correlation matrix of the mobility measures, after discarding 2500 samples as burn-in. Despite the large sample sizes available for each of the 44 months of data, estimating pairwise associations by sample correlation coefficients is not ideal due to the non-Gaussian nature of the univariate marginal distributions of the values of each mobility measure. These marginal distributions show large proportions of callers as having low mobility levels or not moving at all, and also smaller but significant proportions of callers having very high mobility levels. By employing a Gaussian copula, [1] treats the univariate marginal distributions of measures

of mobility as nuisance parameters, thus producing estimates of pairwise correlations with improved statistical properties.

Figures F, G, H and I show the longitudinal pairwise correlations between each of the four existing measures of mobility and the six new measures from groups A, B and C. Figures J, K, L, M, N and O present the longitudinal pairwise associations between each of the six new measures of mobility and the other five measures from groups A, B and C. Since the three groups of measures were defined with respect to the two key dimensions of mobility (frequency of mobility and spatial range), it is important to observe the relationships between each measure with the other measures in the same group (only for groups B and C which contain more than one measure), and also with the measures in the remaining two groups. To this end, we used shades of the same color (green for group B and red for group C) to make it easier to identify correlations with measures in the same group. Correlations with the NT measure from group A are colored in blue. The coloring of groups A, B and C is consistent in all the plots in this section (Figures F–O).

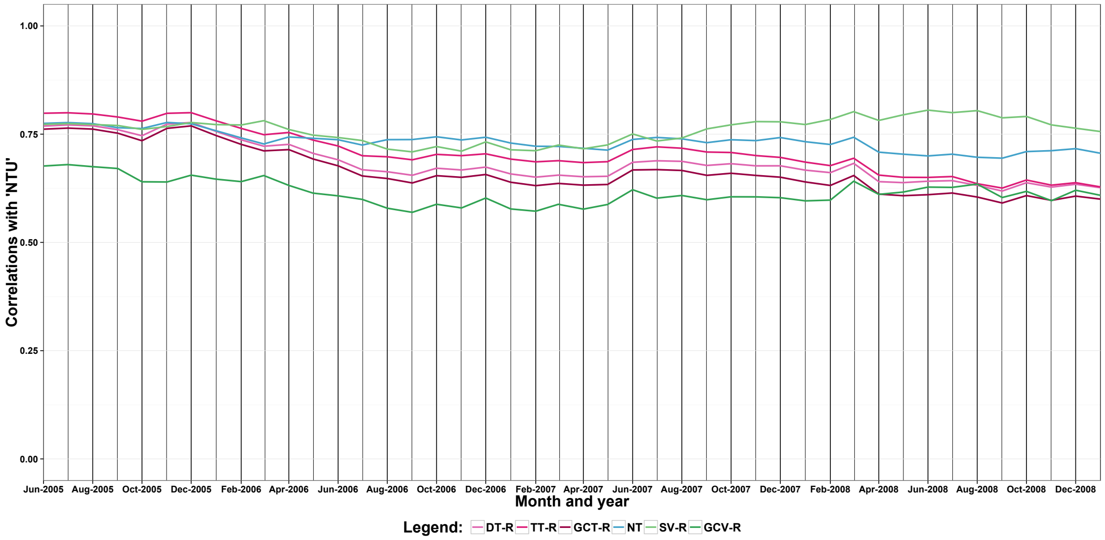

Figure F. Estimated correlations for each of the 44 months of Rwandan CDRs between the existing measure of mobility number of towers used (NTU) and the six measures from Groups A (blue), B (shades of green) and C (shades of red).

Figure F shows that the existing measure of mobility number of towers used (NTU) has comparable correlations with the six new measures. The range of these correlations seem to be larger in 2008 compared to 2005, possibly due to the spatial range and the density of towers being significantly larger in 2008. Recall that tower density influences NTU but not our new measures. Notably, NTU seems to have the strongest longitudinal associations

with the SV-R measure from group B which captures spatial range but does not capture the frequency of mobility. This is not surprising, since NTU also does not capture the frequency of mobility, and the manner in which it captures spatial range is confounded by the varying density of cellular towers. Nevertheless, NTU has the weakest association with the GCV-R measure, also from group B. As GCV-R counts grid cells on quickest road routes and SV-R counts only a subset of these grid cells which are also sites, we can see why NTU would be more similar to SV-R: NTU is based only from places in which calls were made. That said, NTU's correlations with the six new measures are consistent with NTU not being classified in any of the groups A, B and C.

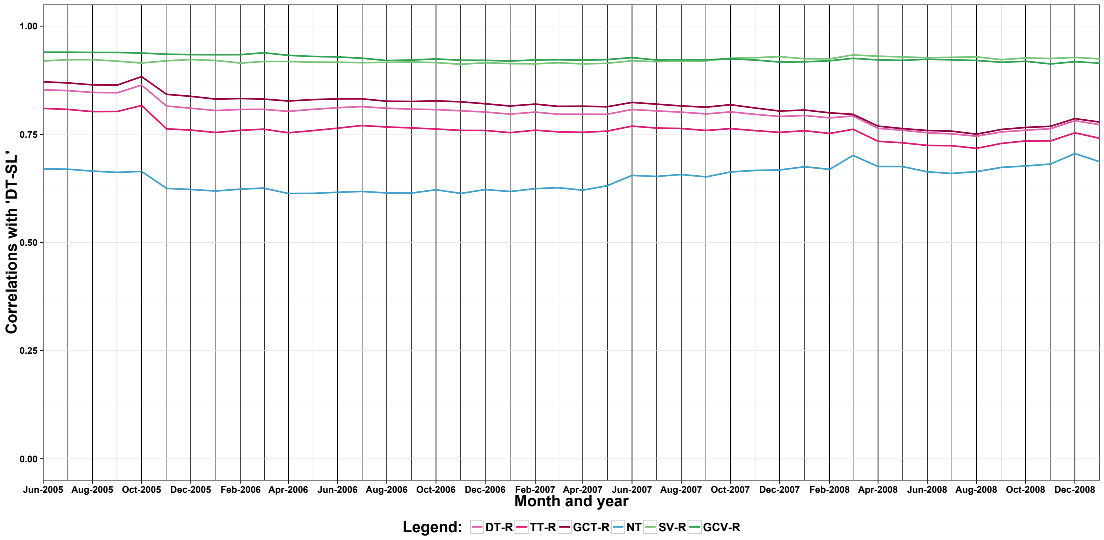

FIGURE G. Estimated correlations for each of the 44 months of Rwandan CDRs between the existing measure of mobility distance traveled (DT-SL) and the six measures from Groups A (blue), B (shades of green) and C (shades of red).

Figures G, H and I show that the existing measures of mobility distance traveled (DT-SL), maximum distance traveled (MDT), and radius of gyration (RoG) share a common pattern of longitudinal associations with the six new measures of mobility: their strongest correlations are with the measures from group B, their second strongest associations are with the measures from group C, and their weakest associations are with the measure from group A. This is precisely as expected in the case of MDT and RoG since both measures capture information related to spatial range, but do not capture any aspect of the frequency of mobility which is key for the measures in groups A and C. However, this pattern of associations is somewhat surprising for DT-SL. We classified this measure in group C because it seemed to capture frequency of mobility and spatial range, and subsequently

we expected to see the largest correlations with the new measures of mobility from group C, especially with distance traveled (DT-R). But there are key differences between DT-SL and DT-R: (i) DT-SL involves movement between cellular towers, while DT-R involves movement between sites; and (ii) DT-SL is the sum of straight line distances, while DT-R is the sum of distances via road travel. The lower longitudinal associations we observe between DT-SL and DT-R emphasize the key differences in the way these two measures are constructed.

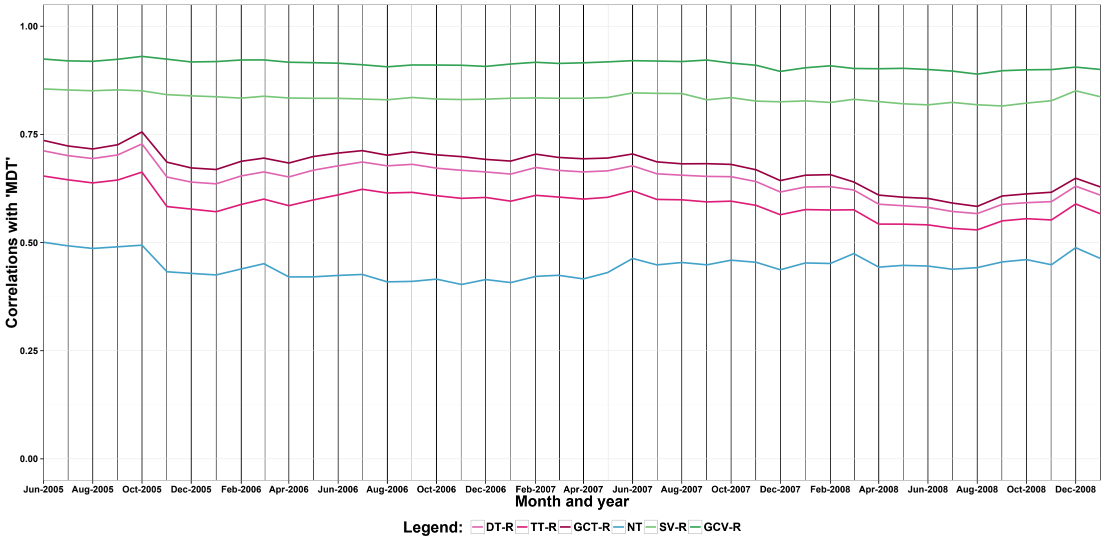

Figure H. Estimated correlations for each of the 44 months of Rwandan CDRs between the existing measure of mobility maximum distance traveled (MDT) and the six measures from Groups A (blue), B (shades of green) and C (shades of red).

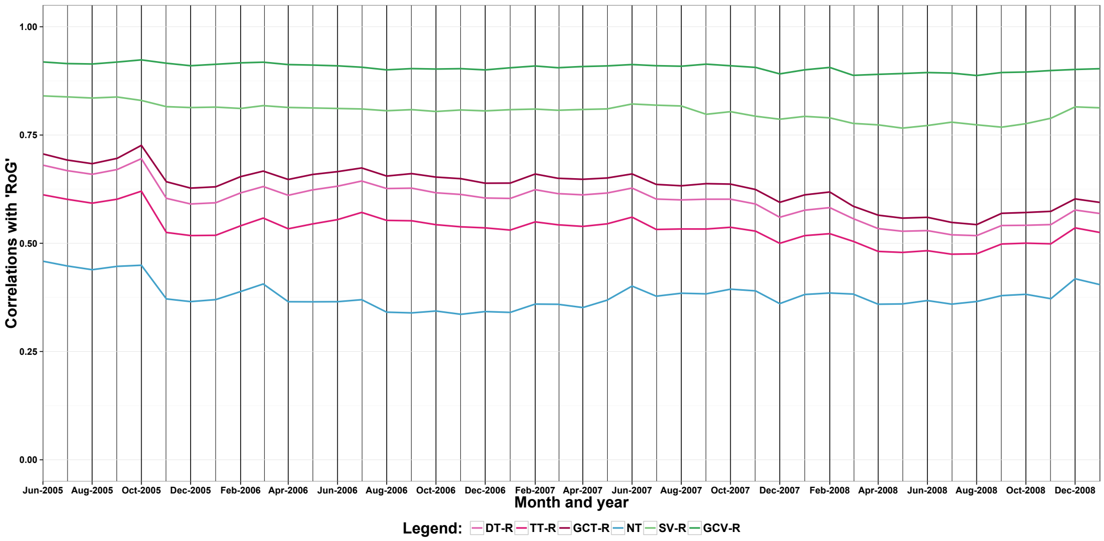

Figure I. Estimated correlations for each of the 44 months of Rwandan CDRs between the existing measure of mobility radius of gyration (RoG) and the six measures from Groups A (blue), B (shades of green) and C (shades of red).

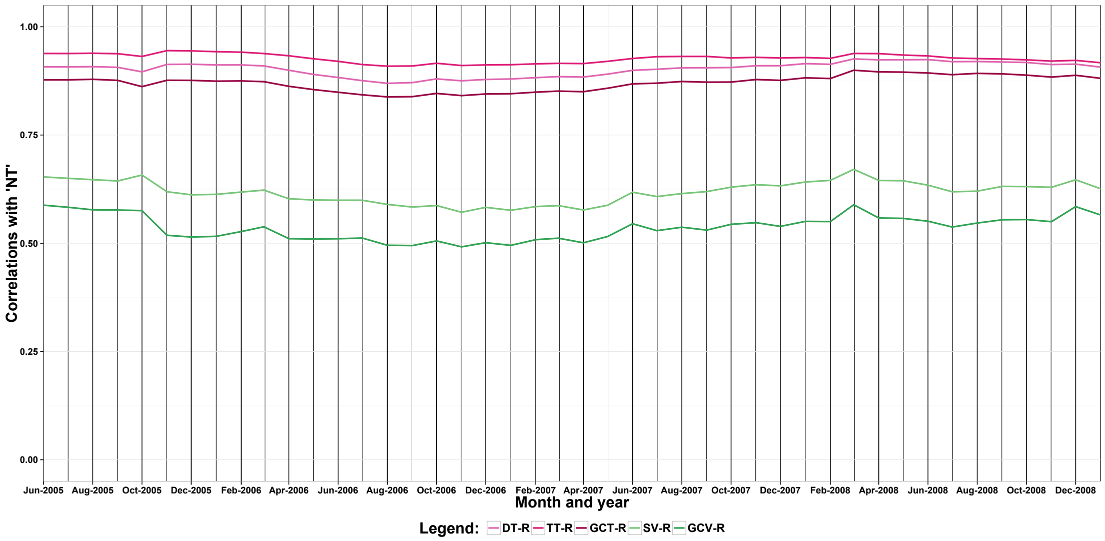

Figure J. Estimated correlations for each of the 44 months of Rwandan CDRs between the new measure of mobility number of trips (NT) which defines group A, and the five measures from Groups B (shades of green) and C (shades of red).

Figure J shows that the new measure of mobility number of trips (NT) which defines group A has the strongest longitudinal associations with the measures from group C, and weaker associations with the measures from group B. This is consistent with our intuition about these measures: NT captures the frequency of mobility as do measures from group C. But NT does not capture spatial range of mobility which explains its lower correlations with the measures from group B.

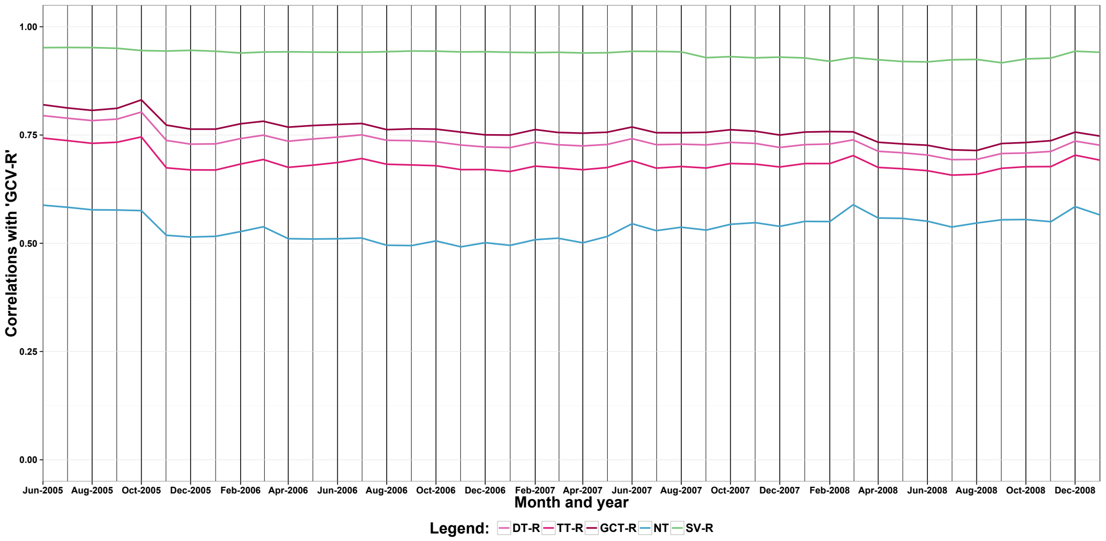

Figure K. Estimated correlations for each of the 44 months of Rwandan CDRs between the new measure of mobility grid cells visited (GCV-R) from group B, and the other measure of mobility from group B (SV-R, green), as well as the four measures from Groups A (blue) and C (shades of red).

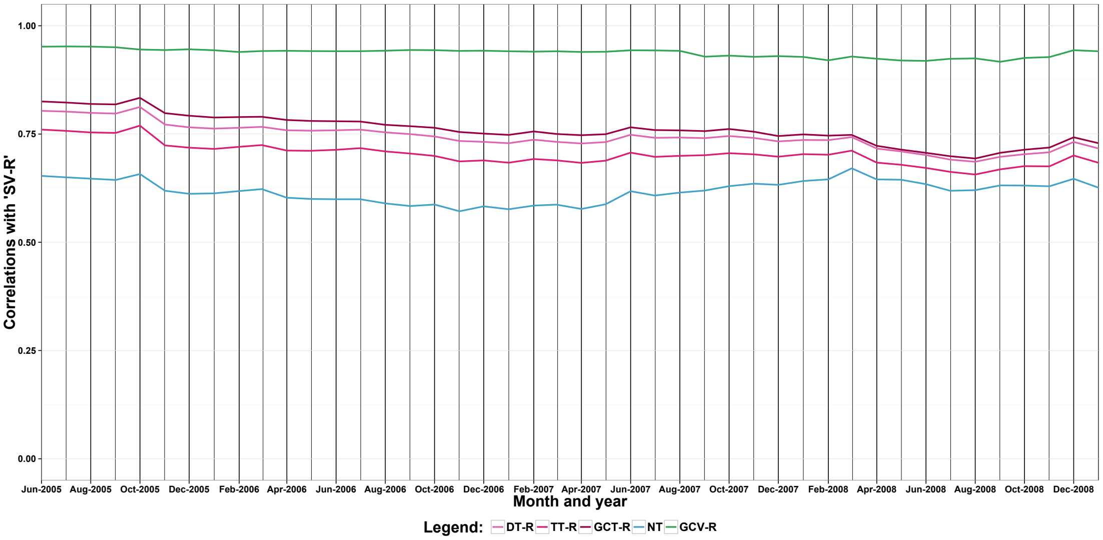

Figure L. Estimated correlations for each of the 44 months of Rwandan CDRs between the new measure of mobility sites visited (SV-R) from group B, and the other measure of mobility from group B (GCV-R, green), as well as the four measures from Groups A (blue) and C (shades of red).

Figures K and L show that the two new measures of mobility from group B have strong longitudinal correlations with each other. Their second strongest longitudinal correlations are with the three measures from group C. This is consistent with our intuition since the five measures from group B and C capture spatial range. Their weakest longitudinal associations are estimated to be with the measure of mobility number of trips (NT) from group A. This is logical since NT captures only the frequency of mobility, while the two measures from group B capture only spatial range.

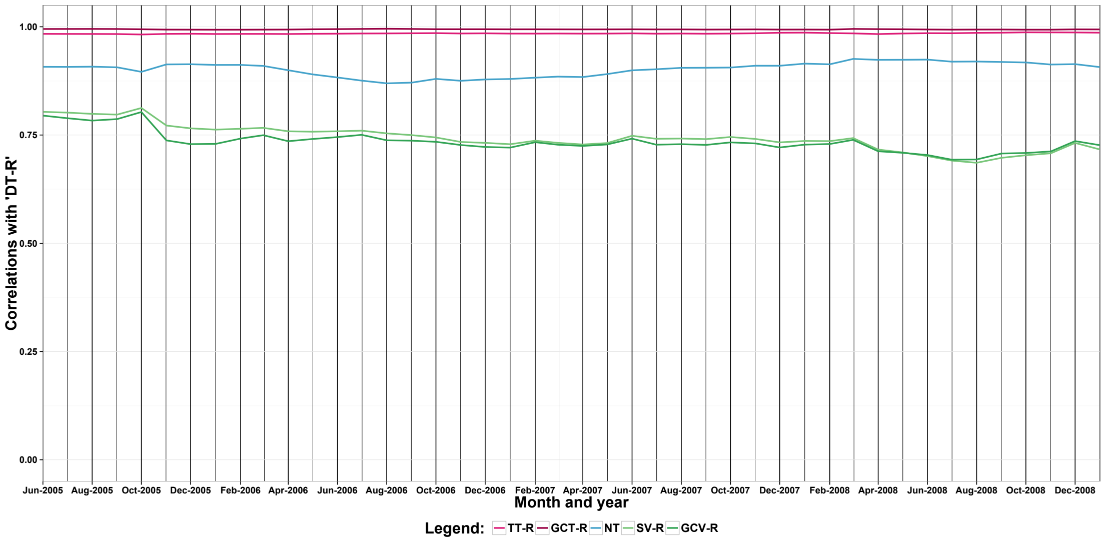

Figure M. Estimated correlations for each of the 44 months of Rwandan CDRs between the new measure of mobility distance traveled (DT-R) from group C, and the other measures of mobility from group C (TT-R and GCT-R, shades of red), as well as the three measures from Groups A (blue) and B (shades of green).

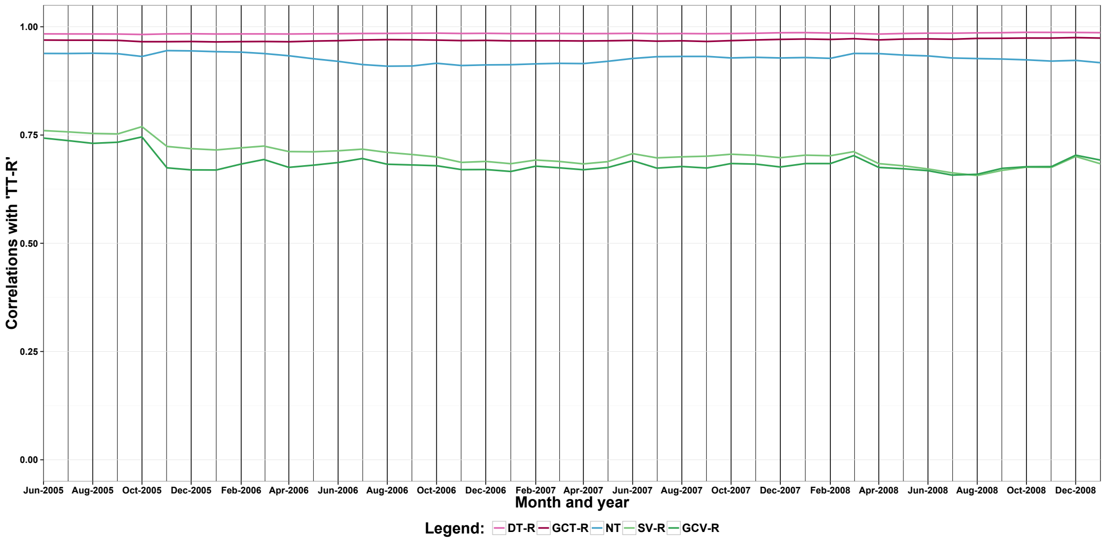

Figure N. Estimated correlations for each of the 44 months of Rwandan CDRs between the new measure of mobility time traveled (TT-R) from group C, and the other measures of mobility from group C (DT-R and GCT-R, shades of red), as well as the three measures from Groups A (blue) and B (shades of green).

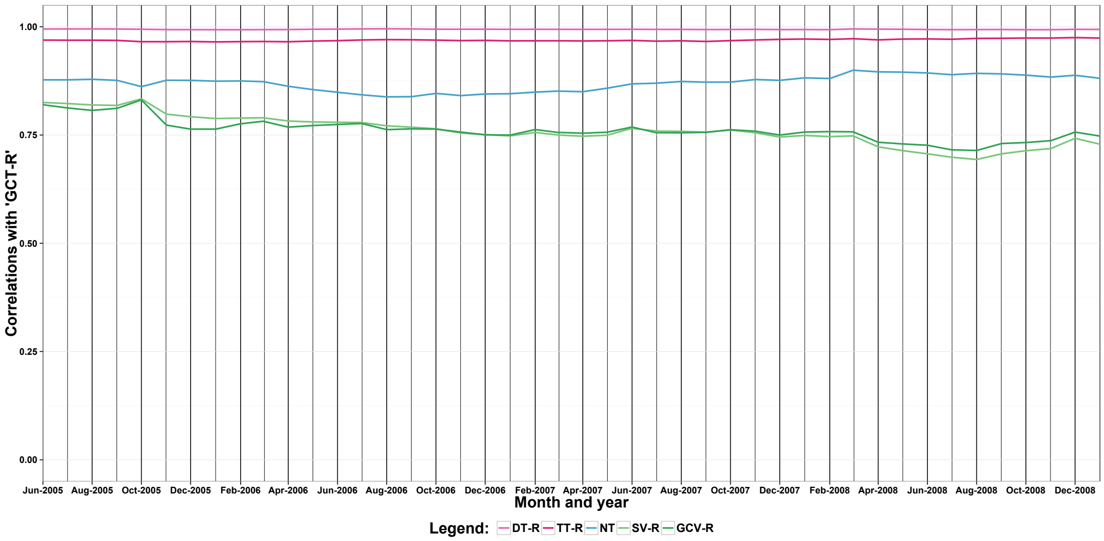

Figure O. Estimated correlations for each of the 44 months of Rwandan CDRs between the new measure of mobility grid cells traveled (GCT-R) from group C, and the other measures of mobility from group C (DT-R and TT-R, shades of red), as well as the three measures from Groups A (blue) and B (shades of green).

Figures M, N and O show that the three new measures of mobility from group C have strong longitudinal associations with each other. Their second strongest longitudinal associations are with the measure of mobility number of trips (NT) from group A. Their weakest longitudinal associations are estimated to be with the two measures from group B. The measures from group B capture spatial range as do the measures from group C, but they do not capture the frequency of mobility. Here it is interesting to see that frequency of mobility is one of the two key dimensions of mobility which leads to larger correlations between the measures in group C (which capture both dimensions) and the measures in groups A and B (which capture only one of these dimensions).

SI6: Categories of callers defined by their mobility with respect to the measures in Groups A, B and C. We identify categories of callers with respect to the six measures of mobility we propose in the main text. A monthly spatiotemporal trajectory of a caller was classified as having low or high mobility with respect to a measure if the corresponding value of trajectory's measure is above or below the median of observed values during that month. For each month, this leads to $2^6 = 64$ segments. The segments which comprise more than 0.05% of the callers in a given month are shown in Tables A, B, C and D. These tables are obtained by cross-classifying spatiotemporal trajectories observed in December 2005, December 2006, December 2007, and December 2008, respectively.

TABLE A. Proportions of callers in categories defined by the six new measures of mobility from Groups A, B and C in December 2005. The measures are evaluated based on the spatiotemporal trajectories of each of the 238,572 persons that used a major cellular phone services provider's Rwandan network during that month. From the $2^6 = 64$ possible categories, only the 37 categories that contain at least 0.05% of the callers from that month are shown.

**Group A**	**Group B**		**Group C**			**Percentage**
NT	GCV-R	SV-R	DT-R	TT-R	GCV-R	
Low	Low	Low	Low	Low	Low	32.06
High	High	High	High	High	High	27.87
High	Low	Low	High	High	High	9.21
Low	High	High	Low	Low	Low	5.08
Low	High	Low	Low	Low	Low	4.59
Low	High	High	High	High	High	4.46
High	Low	Low	Low	Low	Low	3.59
High	High	Low	High	High	High	2.14
High	Low	Low	Low	High	Low	1.94
Low	High	High	High	Low	High	1.87
High	Low	Low	High	High	Low	1.31
High	Low	High	High	High	High	1.02
Low	High	High	Low	Low	High	0.94
Low	High	Low	High	High	High	0.74
Low	High	Low	High	Low	High	0.30
High	High	High	High	Low	High	0.29
High	High	High	Low	Low	Low	0.25
Low	High	Low	Low	Low	High	0.20
High	High	Low	Low	Low	Low	0.16
Low	High	Low	Low	High	Low	0.15
Low	Low	Low	Low	High	Low	0.14
Low	Low	High	Low	Low	Low	0.12
Low	Low	Low	High	High	High	0.12
Low	High	Low	High	High	Low	0.11
High	High	Low	Low	High	Low	0.10
High	High	High	Low	High	Low	0.10
High	High	Low	High	High	Low	0.09
Low	High	High	Low	High	Low	0.08
High	Low	Low	High	Low	High	0.08
Low	Low	Low	Low	Low	High	0.08
High	High	High	High	High	Low	0.08
High	Low	High	Low	High	Low	0.07
High	Low	Low	Low	Low	High	0.07
High	High	Low	High	Low	High	0.06
Low	Low	Low	High	Low	High	0.05
High	Low	High	Low	Low	Low	0.05
High	Low	High	High	High	Low	0.05

Table B. Proportions of callers in categories defined by the six new measures of mobility from Groups A, B and C in December 2006. The measures are evaluated based on the spatiotemporal trajectories of each of the 310,877 persons that used a major cellular phone services provider's Rwandan network during that month. From the $2^6 = 64$ possible categories, only the 39 categories that contain at least 0.05% of the callers from that month are shown.

**Group A**	**Group B**		**Group C**			**Percentage**
NT	GCV-R	SV-R	DT-R	TT-R	GCV-R	
Low	Low	Low	Low	Low	Low	31.11
High	High	High	High	High	High	29.68
High	Low	Low	High	High	High	8.75
Low	High	High	Low	Low	Low	7.97
Low	High	High	High	High	High	3.78
High	Low	Low	Low	Low	Low	3.07
Low	High	Low	Low	Low	Low	2.08
High	Low	Low	Low	High	Low	1.98
Low	High	High	High	Low	High	1.55
High	Low	High	High	High	High	1.40
High	Low	Low	High	High	Low	1.23
High	High	Low	High	High	High	1.06
Low	High	High	Low	Low	High	0.88
High	High	High	Low	Low	Low	0.68
Low	Low	High	Low	Low	Low	0.59
High	High	High	High	Low	High	0.51
Low	High	Low	High	High	High	0.43
Low	Low	Low	High	High	High	0.40
Low	Low	Low	High	Low	High	0.24
High	High	High	Low	High	Low	0.23
Low	Low	Low	Low	Low	High	0.23
High	High	High	High	High	Low	0.23
High	Low	Low	High	Low	High	0.21
Low	Low	Low	Low	High	Low	0.15
High	High	Low	Low	Low	Low	0.12
High	Low	High	Low	Low	Low	0.11
Low	High	Low	High	Low	High	0.11
Low	High	Low	Low	Low	High	0.11
High	Low	High	Low	High	Low	0.10
Low	High	High	Low	High	Low	0.09
High	Low	Low	Low	Low	High	0.08
High	Low	High	High	High	Low	0.08
High	High	High	Low	Low	High	0.07
Low	High	Low	Low	High	Low	0.06
High	High	High	High	Low	Low	0.06
Low	High	High	Low	High	High	0.05
High	Low	Low	High	Low	Low	0.05
Low	Low	Low	Low	High	High	0.05
High	High	High	Low	High	High	0.05

Table C. Proportions of callers in categories defined by the six new measures of mobility from Groups A, B and C in December 2007. The measures are evaluated based on the spatiotemporal trajectories of each of the 552,041 persons that used a major cellular phone services provider's Rwandan network during that month. From the $2^6 = 64$ possible categories, only the 39 categories that contain at least 0.05% of the callers from that month are shown.

**Group A**	**Group B**		**Group C**			**Percentage**
NT	GCV-R	SV-R	DT-R	TT-R	GCV-R	
Low	Low	Low	Low	Low	Low	31.28
High	High	High	High	High	High	28.77
Low	High	High	Low	Low	Low	7.47
High	Low	Low	High	High	High	6.70
High	Low	High	High	High	High	3.53
Low	High	High	High	High	High	3.24
High	Low	Low	Low	Low	Low	2.86
High	High	Low	High	High	High	2.14
Low	High	Low	Low	Low	Low	2.10
High	Low	Low	Low	High	Low	1.38
Low	High	High	High	Low	High	1.31
Low	Low	High	Low	Low	Low	1.20
High	Low	Low	High	High	Low	1.04
Low	High	High	Low	Low	High	0.85
Low	High	Low	High	High	High	0.79
High	High	High	Low	Low	Low	0.64
Low	Low	Low	High	High	High	0.52
High	High	High	High	Low	High	0.49
High	Low	High	Low	Low	Low	0.35
High	Low	High	Low	High	Low	0.30
High	Low	High	High	High	Low	0.26
High	Low	Low	High	Low	High	0.25
High	High	High	High	High	Low	0.23
Low	Low	Low	Low	Low	High	0.23
Low	Low	Low	Low	High	Low	0.21
High	High	High	Low	High	Low	0.21
Low	Low	Low	High	Low	High	0.20
Low	High	Low	Low	Low	High	0.13
Low	High	High	Low	High	Low	0.11
Low	High	Low	High	Low	High	0.10
Low	High	Low	Low	High	Low	0.09
Low	High	Low	Low	High	High	0.09
High	Low	Low	High	Low	Low	0.09
High	High	High	High	Low	Low	0.08
Low	High	High	Low	High	High	0.08
High	Low	Low	Low	Low	High	0.07
High	High	Low	Low	Low	Low	0.07
Low	Low	Low	Low	High	High	0.07
High	High	High	Low	Low	High	0.06

TABLE D. Proportions of callers in categories defined by the six new measures of mobility from Groups A, B and C in December 2008. The measures are evaluated based on the spatiotemporal trajectories of each of the 1,034,431 persons that used a major cellular phone services provider's Rwandan network during that month. From the $2^6 = 64$ possible categories, only the 46 categories that contain at least 0.05% of the callers from that month are shown.

**Group A**	**Group B**		**Group C**			**Percentage**
NT	GCV-R	SV-R	DT-R	TT-R	GCV-R	
Low	Low	Low	Low	Low	Low	31.66
High	High	High	High	High	High	28.69
High	Low	Low	High	High	High	7.28
Low	High	High	Low	Low	Low	7.06
High	Low	High	High	High	High	3.00
Low	High	High	High	High	High	3.00
High	Low	Low	Low	Low	Low	2.90
High	High	Low	High	High	High	2.27
Low	High	Low	Low	Low	Low	2.01
Low	Low	High	Low	Low	Low	1.63
Low	High	High	High	Low	High	1.02
High	Low	Low	High	High	Low	0.88
Low	High	Low	High	High	High	0.88
High	Low	Low	Low	High	Low	0.87
Low	Low	Low	High	High	High	0.84
High	High	High	Low	Low	Low	0.71
Low	High	High	Low	Low	High	0.64
High	High	High	High	Low	High	0.46
High	Low	High	Low	Low	Low	0.39
Low	Low	Low	Low	High	Low	0.35
High	High	High	High	High	Low	0.26
High	Low	High	High	High	Low	0.25
High	Low	Low	High	Low	High	0.24
High	Low	High	Low	High	Low	0.21
Low	Low	Low	High	Low	High	0.20
High	High	High	Low	High	Low	0.16
Low	Low	Low	Low	Low	High	0.16
High	Low	Low	Low	High	High	0.14
Low	High	Low	Low	High	Low	0.14
Low	High	High	Low	High	Low	0.13
Low	High	Low	High	Low	High	0.12
Low	High	High	Low	High	High	0.12
High	Low	Low	Low	Low	High	0.11
Low	High	Low	Low	Low	High	0.11
Low	Low	Low	High	High	Low	0.10
High	Low	Low	High	Low	Low	0.10
Low	High	Low	Low	High	High	0.09
Low	Low	Low	Low	High	High	0.09
High	High	High	Low	Low	High	0.08
High	High	High	High	Low	Low	0.08
Low	Low	High	High	Low	High	0.05
High	High	Low	High	Low	High	0.05
High	High	Low	Low	Low	Low	0.05
Low	High	High	High	High	Low	0.05
Low	Low	High	High	High	High	0.05
High	High	High	Low	High	High	0.05

SI7: Checking the Quality of CDRs with the Measure of Mobility Time Traveled (TT-R)

The measure of mobility time traveled (TT-R) can be used to filter out spatiotemporal trajectories that might have been adversely affected by errors in the cellular service provider's databases of CDRs. Such trajectories can also arise if intruders gain unauthorized access to mobile phones and use them to communicate at the same time as the actual owners. CDRs generated by an intruder and an owner of a mobile phone are saved in the same spatiotemporal trajectory. Measures of mobility for trajectories generated by two or more users of the same phone who aret not located close to each other yield unusually high mobility levels. The TT-R measure can be especially useful in identifying such unusual trajectories that might need to be discarded from population mobility studies.

Here we use the values of TT-R to identify the spatiotemporal trajectories with an average travel time of more than 12 hours per day. We refer to those trajectories with large values of TT-R as unusual, otherwise a trajectory is considered to be normal. In Table E we report the number of normal and the number of unusual trajectories for each of the 44 months of Rwandan CDRs. Despite monthly fluctuations, the percentage of unusual trajectories in a given month never exceed 0.5% of the total number of callers for that month, with only one spike in December 2006. The first and third quartiles, the means and the medians of TT-R appear consistent over time and are not large (500 minutes or 16 travel hours/day). The largest maximum for any month period is large (5000 or 166 travel hours/day), but these extreme outliers represent a small proportion of all the unusual monthly spatiotemporal trajectories, and a very small proportion of the overall dataset.

TABLE E. Identification of the monthly spatiotemporal trajectories with an average travel time of more than 12 hours per day based on the values of the measure of mobility time traveled (TT-R).

Month/ Year	No. of Normal Trajectories	No. of Unusual Trajectories	%	Summary					
				Min.	1st Qu.	Median	Mean	3rd Qu.	Max.
Jun 2005	190634	6	< 0.01	362.5	382.1	422.3	440	439.5	618.8
Jul 2005	200160	5	< 0.01	392.4	416.8	740.4	656.1	848.1	883
Aug 2005	208136	4	< 0.01	380	572.9	703.2	700.4	830.8	1015
Sep 2005	211767	5	< 0.01	404.6	406.5	643.8	614.1	765	850.9
Oct 2005	209705	3	< 0.01	376.7	442	507.3	506.7	571.6	635.9
Nov 2005	226194	113	0.05	360.8	414.3	490.1	541.3	606.1	1217
Dec 2005	238572	138	0.06	374.7	448.9	521.2	600.8	674.1	1755
Jan 2006	244138	123	0.05	373	431	488.8	570.7	637.5	1555
Feb 2006	243243	99	0.04	337.4	375.8	426	497.6	562.1	1266
Mar 2006	249298	79	0.03	372.4	418.9	486.2	549.6	610.8	1508
Apr 2006	253119	123	0.05	364.5	400.2	461.2	531.7	573.7	1675
May 2006	254997	242	0.09	372.1	407.7	465.3	524.2	582.5	1737
Jun 2006	256424	390	0.15	360.5	400.2	461.1	522.2	575.8	3373
Jul 2006	262474	440	0.17	372.1	408.1	462.4	513.5	557.8	3734
Aug 2006	270738	782	0.29	372.1	406.2	465.3	524.5	573.2	4298
Sep 2006	272543	925	0.34	360.2	394	450.9	507.7	558.7	3417
Oct 2006	280419	972	0.35	372.1	413.4	471.7	533.4	585.5	3658
Nov 2006	286481	944	0.33	360	399.5	457.6	509.1	552.3	3490
Dec 2006	310877	1316	0.42	372	409.6	471.7	528	576.5	2510
Jan 2007	320249	1262	0.39	372	409.8	465.8	527.1	570.5	2565
Feb 2007	322645	1083	0.33	336.1	374.3	427.2	486.9	526.4	2838
Mar 2007	338276	1206	0.36	372	414.4	472.9	536.8	580.5	2610
Apr 2007	354874	1112	0.31	360	398.6	453.2	515.7	555.4	2307
May 2007	375186	1071	0.28	372.1	414.3	466.3	532.5	576.2	2563
Jun 2007	406896	915	0.22	360.2	398.7	459	526.2	571.9	2386
Jul 2007	432598	831	0.19	372	409.6	467.4	538.4	581.3	3398
Aug 2007	456355	937	0.2	372.1	413	466.7	543.7	581.5	4037
Sep 2007	472683	746	0.16	360.3	403.1	460.1	530.7	569.9	3566
Oct 2007	500802	1033	0.21	372.2	411	469.9	536.5	584.9	2204
Nov 2007	523857	1250	0.24	360	394.5	452.7	517.9	558.1	2671
Dec 2007	552041	1486	0.27	372	413.8	473.3	541.9	587.7	3235
Jan 2008	583890	1500	0.26	372.1	414.5	481.3	549	587.1	2362
Feb 2008	617341	1528	0.25	348.2	386.5	447	509.7	553.8	2364
Mar 2008	653192	603	0.09	372	399.7	438.6	490	518.3	2279
Apr 2008	675318	657	0.1	360.2	388.7	431.3	497.6	527.4	2331
May 2008	703200	773	0.11	372.2	400.8	445.7	520.1	543.8	2952
Jun 2008	736491	948	0.13	360	392.8	434.3	506.2	529.4	2884
Jul 2008	787210	1050	0.13	372.1	404	451.5	511.6	539.4	2783
Aug 2008	831993	1400	0.17	372	403.3	448.9	522.1	542.9	4851
Sep 2008	864810	1358	0.16	360.1	389.7	434.9	500.4	525.1	4633
Oct 2008	903273	1361	0.15	372	402.1	453.1	517.1	534.6	4479
Nov 2008	941894	1249	0.13	360.1	387.9	431.2	504.2	516.3	5285
Dec 2008	1034431	1728	0.17	372.2	403.8	456.8	530.3	555.9	4186
Jan 2009	1080547	2122	0.2	372	413.4	485.6	561.8	611.2	4147

References

[1] P. D. Hoff, *Extending the rank likelihood for semiparametric copula estimation*, Annals of Applied Statistics, 1 (2007), pp. 265-283.
